# Integrated Multi-Omics Reveals Complementary Luminal and Mucosal Host–Microbiome Interactions Associated with Disease Activity and Phenotype in Paediatric Inflammatory Bowel Disease

**DOI:** 10.3390/microorganisms14092027

**Published:** 2026-09-11

**Authors:** Francesca Toto, Pamela Vernocchi, Paola De Angelis, Sara Isoldi, Salvatore Cucchiara, Laura Stronati, Lorenza Putignani, Federica Del Chierico

**Affiliations:** 1 Research Unit of Microbiome, Bambino Gesù Children’s Hospital, IRCCS, 00165 Rome, Italy; francesca.toto@opbg.net (F.T.); pamela.vernocchi@opbg.net (P.V.); 2 Gastroenterology, Digestive Endoscopy and Nutrition Unit, Bambino Gesù Children’s Hospital, IRCCS, 00165 Rome, Italy; paola.deangelis@opbg.net; 3 Pediatric Gastroenterology Unit, Santobono Pediatric Hospital, 80122 Naples, Italy; isoldi.sara@gmail.com; 4 Pediatric Gastroenterology and Liver Unit, Maternal Child Health Department, Sapienza University of Rome, 00161 Rome, Italy; salvatore.cucchiara@fondazione.uniroma1.it; 5 Department of Molecular Medicine, Sapienza University of Rome, 00161 Rome, Italy; laura.stronati@uniroma1.it; 6 Department of Life Science, Health, and Health Professions, Link Campus University, 00165 Rome, Italy; lorenza.putignani@opbg.net; 7 Unit of Microbiomics and Unit of Microbiome, Bambino Gesù Children’s Hospital, IRCCS, 00146 Rome, Italy

**Keywords:** paediatric inflammatory bowel disease (IBD), Crohn’s disease (CD), ulcerative colitis (UC), gut microbiome, mucosal microbiota, mycobiome, multi-omics integration, microbial ecology, host–microbe interactions, metabolomics

## Abstract

Inflammatory bowel diseases (IBD) arise from complex interactions among the immune system, intestinal microbiota, and host metabolism. However, how different intestinal compartments contribute to disease activity and phenotype in paediatric IBD remains incompletely understood. Children with Crohn’s disease (CD) and ulcerative colitis (UC) were stratified according to disease phenotype and inflammatory activity to distinguish disease-associated signatures from dynamic inflammatory processes. We performed an integrated multi-omics analysis combining luminal and mucosal bacterial and fungal metataxonomy, faecal metabolomics, and microbial- and host-derived biomarkers, including IgA, lysozyme, bile acids, and urinary indican. Luminal bacterial communities largely preserved their ecological structure, while disease activity was associated with coordinated taxonomic, metabolic, and interactional remodelling. These changes were accompanied by alterations in microbial metabolites and host biomarkers consistent with altered fermentation, proteolytic metabolism, and immune–metabolic coupling. In contrast, phenotype-associated ecological differences were more evident in the mucosal compartment, particularly within the fungal community, whereas mucosal bacterial changes were more limited. Together, these findings suggest that luminal and mucosal host–microbiome interactions provide complementary information on inflammatory activity and disease phenotype in paediatric IBD, highlighting the value of integrated multi-omic approaches to investigate compartment-specific host–microbiome interactions in paediatric IBD.

## 1. Introduction

Inflammatory bowel diseases (IBD), including Crohn’s disease (CD) and ulcerative colitis (UC), are chronic relapsing inflammatory disorders resulting from complex interactions among host genetics, environmental exposures, immune dysregulation, and alterations in the intestinal microbiota [1,2,3]. In paediatric patients, these interactions are particularly relevant because early-life immune and microbial development may influence disease onset, progression, and long-term outcomes [4,5]. Despite substantial advances in IBD research, the mechanisms underlying disease activity and phenotype differentiation remain incompletely understood, highlighting the need for integrative approaches capable of investigating host, microbial, and metabolic components simultaneously. Increasing evidence suggests that IBD is better understood as a disruption of intestinal ecosystem organization rather than the consequence of individual pathogenic taxa alone [6,7,8,9,10]. In this context, microbial activity, metabolite production, and host-associated responses may provide insights beyond taxonomic composition alone. Although reductions in microbial diversity have been reported in some IBD cohorts, disease-associated ecological changes do not necessarily involve a complete restructuring of the intestinal ecosystem, but may instead be characterized by selective perturbations of specific microbial groups and functions [11,12,13].

Several host- and microbiome-associated biomarkers have been proposed to characterize intestinal inflammation and microbial ecosystem dynamics [14,15]. Secretory immunoglobulin A (IgA) contributes to immune exclusion, microbial containment, and maintenance of mucosal homeostasis, whereas lysozyme, produced by Paneth cells and macrophages, exerts antimicrobial activity and participates in innate immune defence [16,17,18]. Additional biomarkers, including bile acids and urinary indican, reflect microbial metabolism and intestinal physiological status. Together, these markers provide complementary information regarding inflammatory burden and host-associated responses occurring during IBD [19,20].

Beyond microbial composition, microbial metabolites have emerged as important mediators of host–microbiota interactions and are increasingly recognized as functional indicators of intestinal ecosystem activity [21,22]. Among them, short-chain fatty acids (SCFAs), bile acid derivatives, amino acid metabolites including tryptophan-derived compounds, and other bioactive molecules contribute to epithelial barrier integrity, immune regulation, and microbial ecosystem organization [22,23,24]. Alterations in these metabolic pathways have been associated with IBD, highlighting the value of metabolomic profiling as a functional complement to microbiome analyses [25,26,27,28,29]. Consequently, metabolomic profiling offers a functional perspective that complements microbiome analyses and may help identify disease-associated ecosystem states that are not evident from taxonomic data alone [30]. Recent systems biology studies have therefore increasingly combined microbiome, metabolome, and host-derived measurements to investigate IBD at an ecosystem level [31,32,33]. Within this framework, network-based approaches enable the identification of coordinated microbial and metabolic associations that may contribute to disease-associated ecosystem organization [34,35]. An additional layer of complexity arises from the spatial organization of the intestinal ecosystem. Luminal and mucosal microbial communities occupy distinct ecological niches characterized by different environmental conditions and host interactions [36,37]. While faecal microbiota has been extensively investigated in IBD, mucosal-associated bacterial communities and the intestinal mycobiome remain comparatively understudied despite their close proximity to the epithelial surface and immune system [38,39]. Emerging evidence suggests that fungal populations may contribute to mucosal inflammation and barrier dysfunction, highlighting the importance of considering multi-kingdom microbial communities when investigating intestinal disease [20,30,40].

Despite growing interest in microbiome-based biomarkers and multi-omic integration, the relationships among luminal microbiota, mucosal bacterial and fungal communities, microbial metabolites, and host-associated biomarkers remain insufficiently characterized in paediatric IBD. Furthermore, it remains unclear whether luminal and mucosal compartments provide overlapping or complementary information regarding inflammatory activity and disease phenotype.

To address this knowledge gap, we integrated faecal 16S rRNA microbiome profiling, faecal metabolomics, mucosal (ileal biopsy) bacterial and fungal sequencing, and host- and microbial-associated biomarkers, including secretory IgA, lysozyme, bile acids, and urinary indican. By combining microbial ecology, metabolic profiles, and host-associated responses within a systems framework, we aimed to investigate how luminal and mucosal ecosystems differentially reflect inflammatory activity and disease phenotype in paediatric IBD.

## 2. Materials and Methods

### 2.1. Study Population

Paediatric patients diagnosed with IBD according to the Porto diagnostic criteria [41] were prospectively enrolled at the Paediatric Gastroenterology and Liver Unit of Sapienza University of Rome, Italy [42]. Eligible participants were aged ≤18 years and had not received antibiotic or probiotic treatments within the two months preceding sample collection. Disease activity was assessed using validated paediatric clinical indices, namely the Paediatric Crohn’s Disease Activity Index (PCDAI) for CD and the Paediatric Ulcerative Colitis Activity Index (PUCAI) for UC. Active disease was defined by PCDAI or PUCAI scores greater than 10 [20]. Endoscopic scores (SES-CD, Simple Endoscopic Score for Crohn’s Disease) and Matts score [43]) were reported. All participants followed a standardized dietary regimen comparable to a Mediterranean-style diet. The study was conducted in accordance with the Declaration of Helsinki and received approval from the Institutional Ethics Committees of Sapienza University (4317, prot. n. 240/17) and of Bambino Gesù Children’s Hospital, (Rome, Italy) (prot. n. 2996_OPBG_2022). Written informed consent was obtained from all parents or legal guardians before enrolment.

### 2.2. Mucosal and Faecal Sampling

During ileocolonoscopy, non-inflamed ileal biopsies were obtained from all patients and immediately stored at −80 °C. To minimize the potential confounding effect of anatomical sampling site, ileal biopsies were collected from macroscopically non-inflamed mucosa in all participants, irrespective of disease phenotype. This standardized sampling strategy was adopted to enable direct comparison of the ileal mucosal ecosystem between CD and UC, rather than to characterize the primary site of inflammation in UC.

Fresh faecal samples were collected from IBD patients the day before endoscopy and stored at −20 °C immediately after collection. After collection, samples were transported at controlled temperature to the Microbiome Research Unit of the Bambino Gesù Children’s Hospital, and stored at −80 °C until DNA extraction.

### 2.3. DNA Extraction and Sequencing

Bacterial and fungal DNA extraction from mucosal biopsies and faecal samples was performed as previously described [30,40,44]. For faecal samples, DNA was extracted from 200 mg of stool using the QIAamp Fast DNA Stool Mini Kit (Qiagen, Hilden, Germany). Mucosal biopsies were processed using the EZ1 DNA Tissue Kit in combination with the Qiagen EZ1 Advanced XL instrument, following the manufacturer’s protocols. For fungal analysis, the same biopsy samples underwent a preliminary fungal lysis step by incubation at 37 °C for 30 min under agitation in lysis buffer (50 mM Tris [pH 7.5], 10 mM EDTA, 28 mM 2-mercaptoethanol, and 10 U/mL lyticase; Sigma-Aldrich, St. Louis, MO, USA), before the DNA extraction by the same procedure described for bacterial analysis.

### 2.4. Microbiota and Mycobiota Sequencing

Bacterial community profiling was performed by 16S rRNA gene amplicon sequencing targeting the V3–V4 hypervariable region (~460 bp) using primers described in the Illumina MiSeq rRNA Amplicon Sequencing protocol (Illumina, San Diego, CA, USA) [30].

Fungal communities were characterized by amplification of the ITS2 region (~350 bp) using the primers 5′–GTGARTCATCGAATCTTT–3′ and 5′–GATATGCTTAAGTTCAGCGGGT–3′ [30,40,45]. PCR amplification conditions were: 94 °C for 2 min, followed by 35 cycles of 94 °C for 15 s, 52 °C for 30 s, and 72 °C for 45 s. PCR products were purified using CleanNGS magnetic beads (CleanNA, Waddinxveen, The Netherlands) prior to a second 15-cycle PCR, using Illumina-adapted ITS2 primers. Indexing was subsequently performed using the Nextera XT Index Kit (Illumina, Inc., San Diego, CA, USA).

Both bacterial and fungal libraries were quantified with the Quant-iT™ PicoGreen^®^ dsDNA Assay Kit (Invitrogen, Thermo Fisher Scientific, Waltham, MA, USA), pooled, normalized to a concentration of 4 nM, and sequenced on the Illumina MiSeq platform, generating paired-end reads (2 × 250 bp) according to the manufacturer’s instructions.

For all amplification steps, positive and negative controls were included to detect possible internal or external contamination. No amplification was detected in the negative controls; therefore, no contaminant ASVs were identified or removed prior to downstream analyses.

### 2.5. Bioinformatic and Statistical Analysis

Raw sequencing data (FASTQ files), obtained from the Illumina MiSeq platform, were processed using the Galaxy platform (https://usegalaxy.org/, accessed on 2 March 2026) through standardized microbiome analysis workflows [46] with the DADA2 pipeline [47], following analytical steps broadly consistent with those implemented in QIIME 2. In particular, adapter and primer sequences as well as low-quality bases were removed using Cutadapt [48], generating high-quality reads that were sorted to ensure consistency across samples and paired-end libraries. Sequence quality was assessed using FastQC [49] and MultiQC [50] and read quality profiles were inspected using the DADA2 function *plotQualityProfile* prior to filtering to guide parameter selection. Reads were then filtered and trimmed using the *filterAndTrim* function with dataset-specific parameters (truncLen, maxEE, truncQ), and filtering performance was verified post hoc. Error rates were independently estimated for forward and reverse reads using *learnErrors*. Sequencing quality control metrics, including raw sequencing depth, high-quality reads retained after filtering, and the number of retained ASVs for each dataset, are reported in Supplementary Table S1.

Amplicon sequence variants (ASVs) were inferred using the *dada* algorithm based on the learned error models. Paired-end reads were merged with *mergePairs*, and an ASV table was generated using *makeSequenceTable*, representing ASV abundances across samples. Chimeric sequences were removed de novo using *removeBimeraDenovo*. Taxonomic assignment was performed using the Greengenes2 reference database (2024.09 release) [51] for 16S rRNA gene sequences and the UNITE database v10.0 for ITS2 sequences [52], applying the *assignTaxonomy* function followed by species-level annotation with *addSpecies*. A phyloseq object was generated in R (version 4.5.2) within RStudio (version 2026.01.1) using the phyloseq package to integrate ASV, taxonomy, and sample metadata. Low-prevalence ASVs were filtered by retaining only those detected in at least 25% of the samples prior to normalization and downstream statistical analyses. This filtering step was applied to reduce the influence of low-frequency features while preserving robust ecological signals.

Data were normalized by cumulative sum scaling (CSS) to account for the compositional nature of microbiome data [53,54]. Downstream statistical analyses were performed using the validated implementations available in MicrobiomeAnalyst 2.0 [55]. To assess within-sample diversity (alpha diversity), the Shannon and Chao1 indexes were calculated, and statistical significance was determined using the Kruskal–Wallis test. To evaluate differences between groups (beta diversity), Bray–Curtis and Unweighted UniFrac distances were calculated and visualized via Principal Coordinate Analysis (PCoA); statistical significance was assessed using Permutational Multivariate Analysis of Variance (PERMANOVA). Differential abundance analysis of taxa was performed using edgeR with a significance threshold of false discovery rate (FDR) < 0.05 (Benjamini–Hochberg test). Microbial co-occurrence networks within bacterial and fungal communities were inferred using SparCC, a method specifically developed for compositional microbiome data. Significant associations were retained based on SparCC correlation coefficients. Data visualization was performed through the MicrobiomeAnalyst graphical interface [55].

### 2.6. Intestinal Permeability, Mucosal Immune Activation, and Metabolic Dysbiosis Markers

Markers of intestinal permeability, mucosal immune activation, and metabolic dysbiosis were measured using commercial ELISA kits.

Zonulin (Zpn), IgA (sIgA) and lysozyme faecal levels were quantified using ELISA kits (Immundiagnostik AG, Bensheim, Germany), according to the manufacturer’s instructions. Absorbance was measured at 450 nm using a microplate reader.

Faecal bile acids were detected by a fluorometric test (Sigma-Aldrich, Merck, St. Louis, MO, USA) with an excitation wavelength of 530 nm and an emission wavelength of 585 nm using a microplate reader.

Urinary indican (indoxyl sulfate) levels were measured using the QuantyChrom™ Indican Assay Kit (BioAssay Systems, Hayward, CA, USA) following the manufacturer’s protocol. Absorbance was read at 480 nm using a microplate reader.

### 2.7. Faecal Metabolomics

Stool samples were analysed for volatile organic compounds (VOCs) using gas chromatography-mass spectrometry (GC-MS) coupled with solid-phase microextraction (SPME), as previously described [30,56]. VOCs were extracted using an 85 μm CAR-PDMS fiber (Supelco) exposed to each sample for 45 min, followed by thermal desorption in the GC injector for 10 min. Analyses were performed on an Agilent 7890B GC system coupled to a 5977A mass detector in electron impact mode, equipped with a DB-HeavyWaX capillary column.

Compound identification required a spectral match ≥80%, with manual verification when needed. Quantification was performed using 4-methyl-2-pentanol (400 ppm) as an internal standard, expressing results as mg/kg. Quality control samples were periodically analysed to monitor instrument stability and analytical reproducibility. VOC identification was based on comparison with the NIST mass spectral library and confirmed, when necessary, by matching retention times with reference standards. Metabolites were quantified as relative abundances normalized to the internal standard and therefore should be considered semi-quantitative measurements.

Metabolomics data were analysed using the validated statistical implementations available in the web-based platform MetaboAnalyst 6.0 [57]. Prior to analysis, metabolite abundance data were normalized by sum, log-transformed, and auto-scaled. Multivariate analyses included principal component analysis (PCA), partial least squares discriminant analysis (PLS-DA), and correlation network reconstruction using SparCC [57,58]. Differential metabolite analysis was performed using the limma (Linear Models for MicroArray Data) statistical framework [59]. Statistical significance was determined using the Benjamini–Hochberg FDR correction with an adjusted *p*-value < 0.05. Differentially abundant metabolites were additionally filtered according to absolute log2 fold change (log2FC) and visualized using Volcano Plots (https://huygens.science.uva.nl/VolcaNoseR/, accessed on 2 March 2026).

### 2.8. Statistical Strategy and Integrative Analysis

Clinical and demographical continuous variables were expressed as mean ± standard deviation (SD) (Table 1). No a priori sample size calculation was performed due to the exploratory multi-omic design. Group comparisons were performed using Mann–Whitney U test or Student’s t-test for independent samples. Fisher’s Exact Test was used for association analysis between categorical variables.

To evaluate the potential influence of clinical confounders on the principal host-derived biomarkers, metabolites and representative taxa, additional multivariable linear regression analyses were performed using IBM SPSS Statistics (IBM SPSS Statistic 21; Armonk, NY, USA). Individual biomarkers, metabolites and taxa identified in the primary differential analyses were considered as dependent variables. Age, disease duration, biological treatment, 5-AZA treatment, disease activity, and disease phenotype were included simultaneously as independent variables. Regression coefficients and associated *p*-values were used to assess whether the observed associations remained independent of the main clinical covariates.

Correlations between taxa, metabolites, clinical indices, and biomarkers were evaluated using Pearson’s correlation coefficient.

Biomarkers comparisons were performed by Mann–Whitney U test and Linear Regression Analysis and graphed by violin plots. Due to skewed distributions, biomarker values were log-transformed prior to analysis (GraphPad Prism 10; Boston, MA, USA). A *p*-value < 0.05 was considered statistically significant. The biomarker panel consisted of predefined hypothesis-driven variables; therefore, no multiple-testing correction was applied to these analyses.

Venn diagram was calculated by the count of the ASVs included exclusively in “luminal”, those included exclusively in “mucosal” and those in common between “luminal” and “mucosal” [60].

Jaccard similarity (luminal vs. mucosal) was calculated with the formula [61]

$$J=\frac{shared\;}{luminal+mucosa-shared}$$

shared = ASVs between luminal and mucosal.

Sørensen coefficient was calculated with the formula [62]

$$S=\frac{2*shared}{\left(luminal+mucosal\right)}$$

shared = ASVs between luminal and mucosal.

For ecological interpretation, bacterial genera were additionally classified into functional categories (including SCFA producers, mucin degraders, commensals, opportunistic bacteria, inflammation-associated taxa, and mucus-associated bacteria) according to published literature.

### 2.9. Network Analysis (Gephi)

Correlation networks integrating microbial taxa, fungal taxa, metabolites, and host-derived biomarkers were generated using Spearman’s rank correlation analysis. Only significant correlations (|ρ| ≥ 0.7 and *p* < 0.05) were retained and visualized using Gephi software (version 0.10.1, Gephi Consortium, Paris, France) with the ForceAtlas2 layout algorithm [63]. Nodes represent metabolites and microbial taxa or microbial and fungal taxa, while edges represent pairwise correlations, with edge weights corresponding to correlation coefficients (ρ). Only significant associations (|ρ| ≥ 0.7 and *p* < 0.05) were retained. 

## 3. Results

### 3.1. Characteristics of the Study Population

A total of 46 paediatric patients with inflammatory bowel disease were included, comprising 24 with UC and 22 with CD. Sixteen patients (34.8%) presented with clinically active disease, while 30 (65.2%) were in remission or had inactive disease at the time of sampling (Table 1).

No significant differences in disease activity were observed between UC and CD patients. Biological therapy was significantly more frequent among CD patients (45.5%) compared with UC patients (12.5%, *p* = 0.01), whereas 5-aminosalicylic acid (5-ASA) therapy was more commonly used in UC (50.0% vs. 22.7%, *p* = 0.04). Ileal involvement predominated in CD, whereas distal and extensive colonic disease were mainly observed in UC.

When patients were stratified according to disease activity, both clinical activity scores (PCDAI/PUCAI) and endoscopic scores (SES-CD/Matts score) were significantly higher in patients with active disease (*p* < 0.001). No significant differences were observed between active and inactive patients regarding age, body mass index (BMI), disease duration, gender distribution, disease type, or treatment exposure.

### 3.2. Faecal and Urinary Biomarkers Indicate Activity-Dependent Inflammatory Signals

To establish the host inflammatory and metabolic context before microbiome analyses, faecal and urinary biomarkers were first evaluated according to both disease phenotype and inflammatory activity, stratifying patients into four clinically relevant groups: CD inactive, CD active, UC inactive, and UC active. This grouping enabled the identification of metabolic alterations associated with disease type while simultaneously capturing activity-related changes.

**Figure 1:**
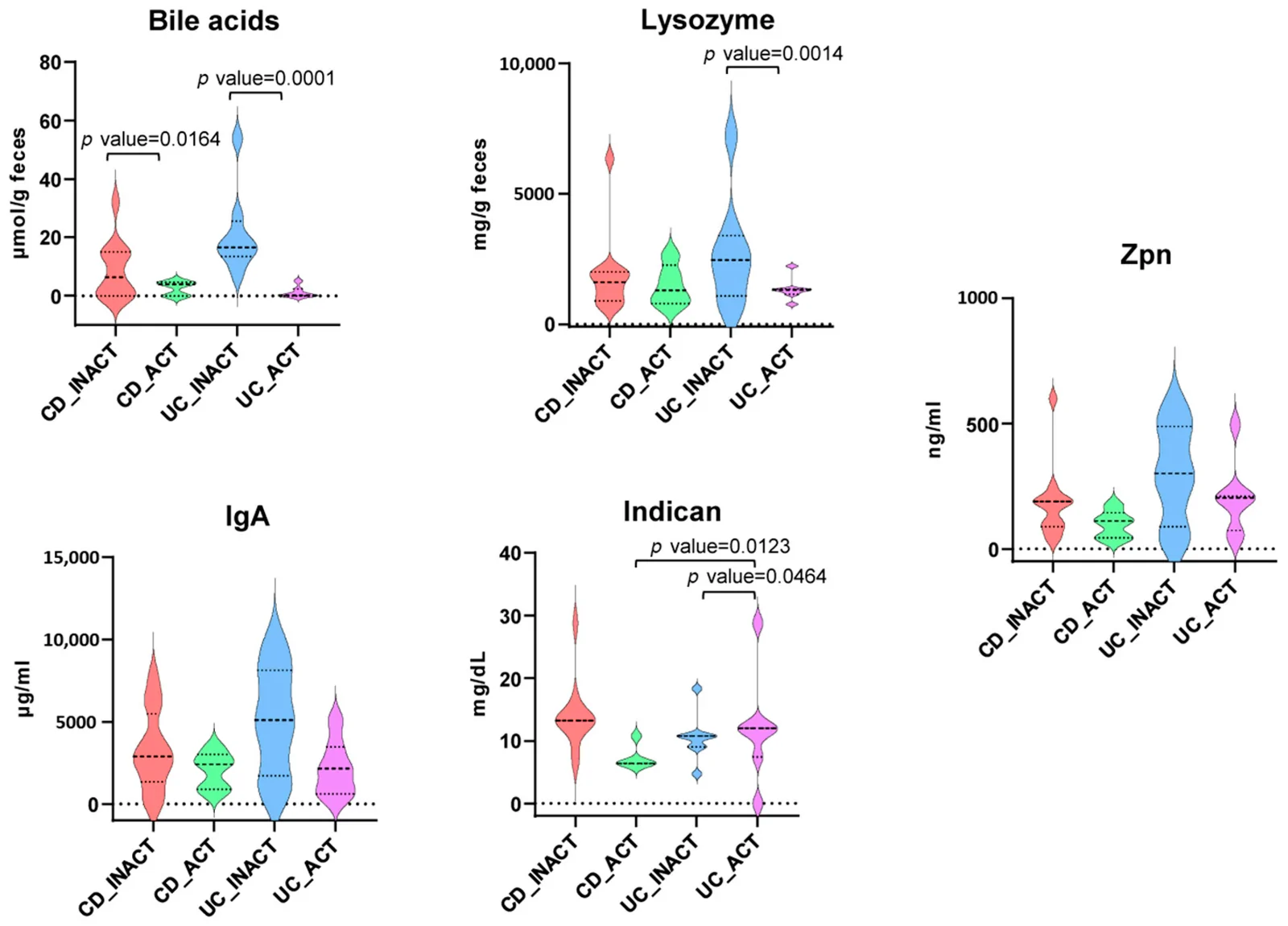
Host-derived faecal and urinary biomarkers reflect inflammatory activity in paediatric IBD. Violin plots showing raw values of faecal lysozyme, secretory IgA, zonulin (Zpn), total bile acids, and urinary indican levels across four clinical groups: UC inactive, UC active, CD inactive, and CD active. Biomarkers were analysed to evaluate immune activation, barrier integrity, and microbial metabolic dysbiosis. Statistical comparisons were performed using the Mann–Whitney U test; Significant comparisons (*p* ≤ 0.05) are indicated.

Faecal bile acids were significantly higher in inactive compared with active disease in both CD and UC (*p* = 0.0164 and *p* = 0.0001, respectively) (Figure 1, Supplementary Table S2).

Lysozyme levels were also increased in UC inactive compared with UC active patients (*p* = 0.0014). Urinary indican was elevated in UC active compared with UC inactive and CD active (*p* = 0.0464 and *p* = 0.0123, respectively). Zonulin and IgA showed no group differences. Descriptive statistics for all biomarkers are reported in Supplementary Table S2. Correlation analyses identified coordinated immune–metabolic signals. Bile acids correlated with lysozyme (ρ = 0.525, *p* = 0.002) and with IgA (ρ = 0.621 and *p* < 0.001), while lysozyme strongly correlated with IgA (ρ = 0.584 and *p* < 0.001), suggesting linked mucosal immune activation. No other significant associations were found among biomarkers. None of the biomarkers correlated with clinical activity indices, indicating partial independence between biochemical signals and clinical scoring systems.

Linear regression analysis confirmed the significant association between bile acids and lysozyme, and between lysozyme and IgA, supporting coordinated mucosal immune activation. Conversely, zonulin was significantly associated with urinary indican, suggesting a link between intestinal permeability and microbial metabolite production (Table 2).

### 3.3. Luminal Ecosystem Shows Functional Adaptation Without Major Structural Disruption

**Figure 2:**
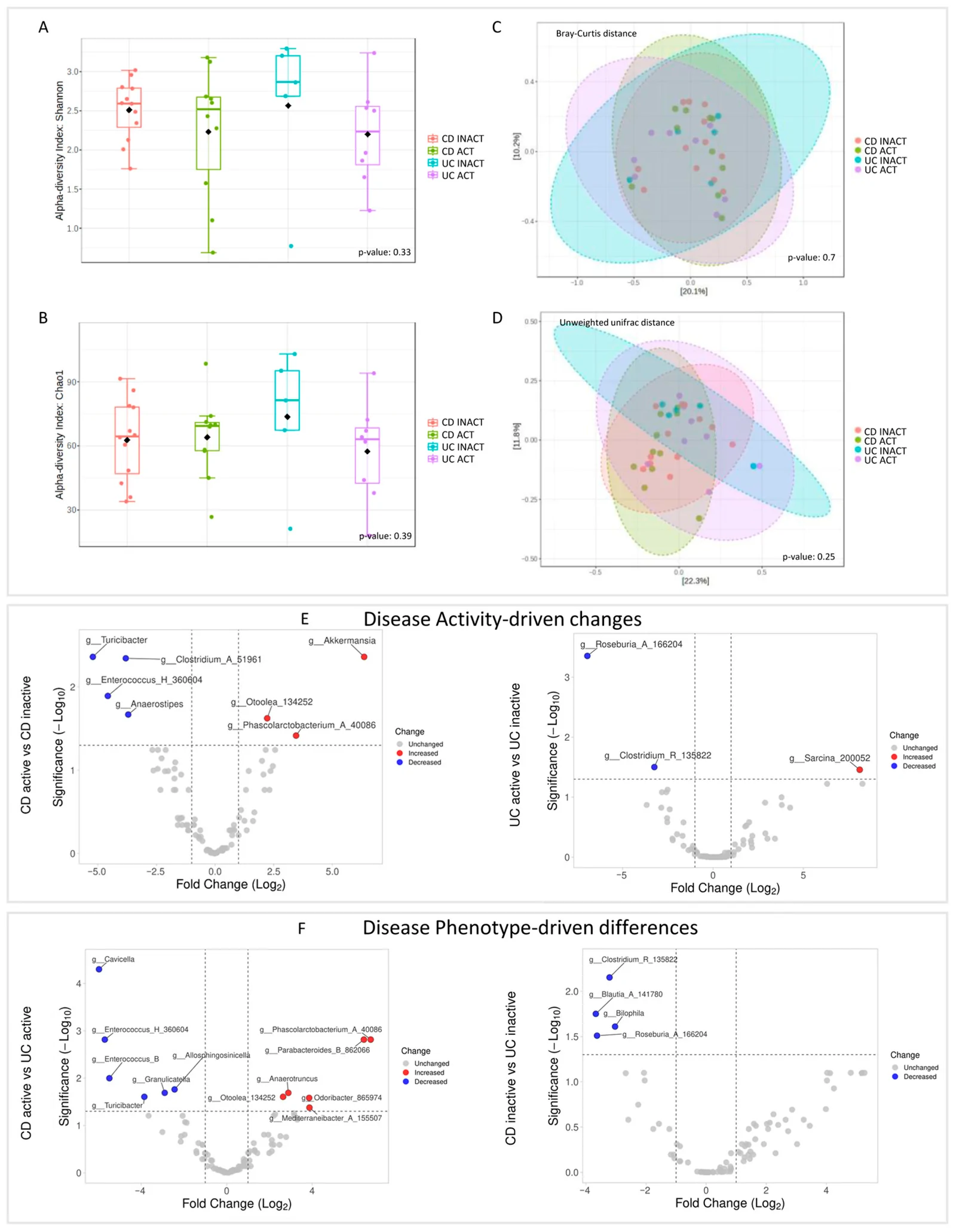
Luminal bacterial microbiota shows preserved diversity with selective taxonomic shifts. (**A**,**B**) Alpha diversity measured by Shannon and Chao1 indices across CD inactive, CD active, UC inactive, and UC active groups, showing no significant differences (*p* = 0.33 and *p* = 0.39). Black diamonds indicate the group mean. (**C**,**D**) Principal coordinate analysis (PCoA) based on Bray–Curtis and unweighted UniFrac distances demonstrating absence of global community segregation by disease activity or phenotype (*p* = 0.70 and *p* = 0.25). (**E**) Volcano plots illustrating differential abundance associated with disease activity (CD active vs. CD inactive; UC active vs. UC inactive). (**F**) Volcano plots illustrating phenotype-associated differences (CD vs. UC in active and inactive states). Dashed lines indicate statistical significance thresholds.

In faecal samples, alpha diversity did not differ across groups (Figure 2A,B).

Beta diversity analyses revealed no clustering by disease type or activity (PERMANOVA *p* = 0.70 and *p* = 0.25), indicating preservation of global ecosystem structure (Figure 2C,D). Despite stable community architecture, differential abundance analysis identified activity-associated taxa. Active CD showed decreased *Turicibacter*, *Clostridium*_A_51961, *Enterococcus*_H_360604, and *Anaerostipes*, together with enrichment of *Akkermansia*, *Otoolea*_134252, and *Phascolarctobacterium*_A_40086. Active UC was characterized by increased *Sarcina*_200052 and decreased *Roseburia*_A_166204 and *Clostridium*_R_135822 (Figure 2E).

Direct comparison of active diseases revealed enrichment in CD of *Phascolarctobacterium*_A_40086, *Parabacteroides*_B_862066, *Anaerotruncus*, *Otoolea*_134252, *Odoribacter*_865974, and *Mediterraneibacter*_A_155507, whereas active UC samples were enriched in *Cavicella*, *Enterococcus* lineages, *Allosphingosinicella*, *Granulicatella*, and *Turicibacter*. During remission, CD showed reduced *Clostridium*_R_135822, *Blautia*_A_141780, *Bilophila*, and *Roseburia*_A_166204 compared with UC (Figure 2F).

Correlation analyses identified structured associations among bacterial taxa despite taxonomic differences between disease groups (Figure S1).

To determine whether these taxonomic changes were accompanied by functional alterations, faecal metabolomic profiles were subsequently investigated.

**Figure 3:**
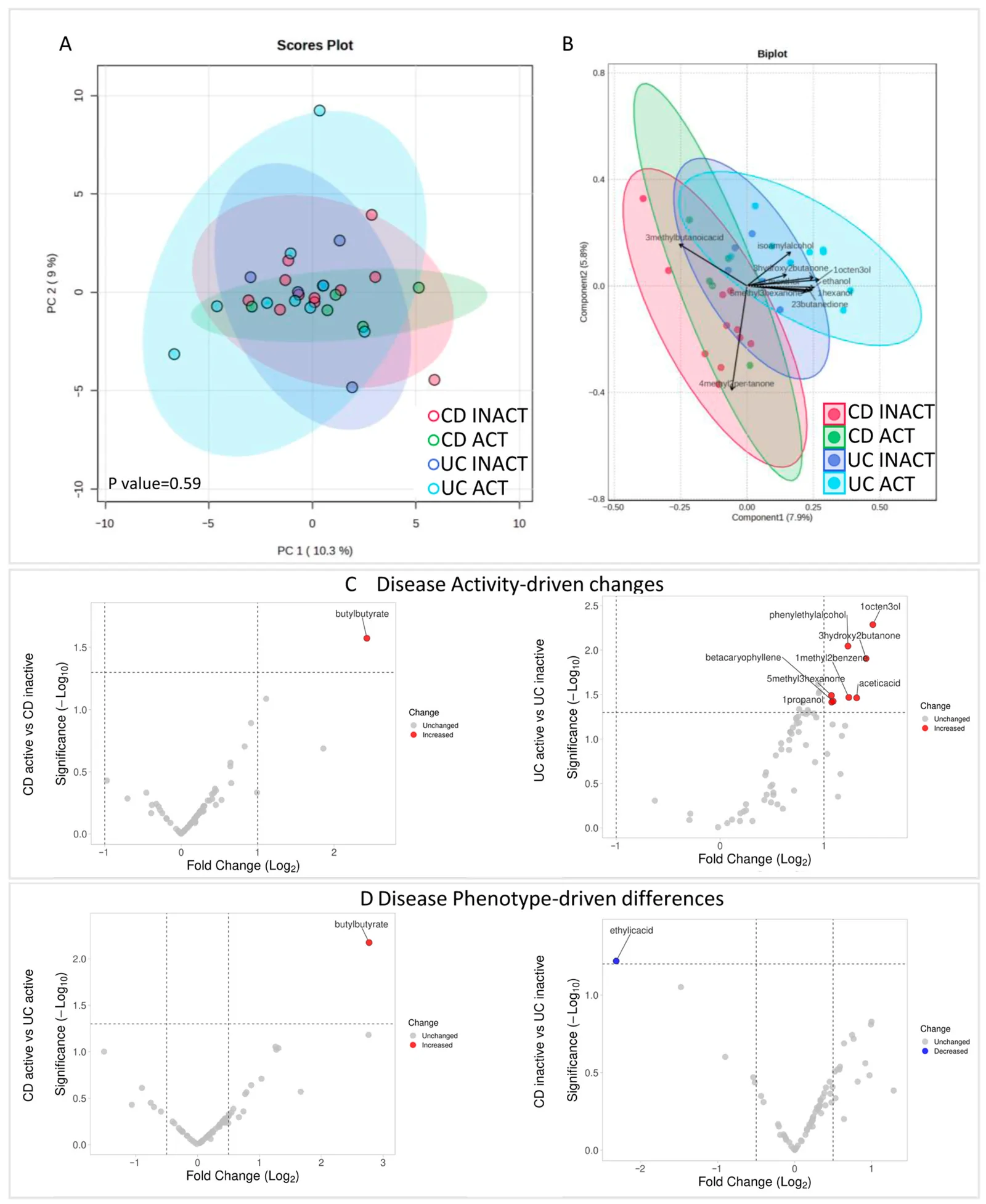
Faecal metabolomic profiling reveals functional remodelling without global compositional separation. (**A**) Principal component analysis (PCA) score plot showing distribution of faecal metabolomic profiles from CD and UC patients stratified by disease activity, demonstrating overlapping metabolic landscapes (PERMANOVA *p* = 0.59). (**B**) PCA biplot indicating metabolites contributing most strongly to variance along principal components. (**C**,**D**) Volcano plots showing metabolites associated with disease activity (CD active vs. CD inactive; UC active vs. UC inactive). Dashed lines indicate statistical significance thresholds.

Multivariate metabolomic analyses showed extensive overlap among groups, with no significant separation in PCA or PLS-DA models (*p* = 0.59) (Figure 3), indicating conserved global metabolomic composition.

Targeted differential analysis nevertheless identified activity-related metabolic changes. Active CD exhibited increased butyl butyrate. Active UC showed enrichment of several alcohols, ketones, aromatic compounds, and acetic acid, suggesting intensified fermentation and lipid oxidation pathways. Butyl butyrate remained higher in CD compared with UC during active disease, while ethyl acetic acid was reduced in CD remission relative to UC.

To assess the potential influence of clinical confounders, additional multivariable linear regression analyses were performed on the principal host-derived biomarkers, metabolites and representative bacterial taxa (Supplementary Table S3). Disease activity remained independently associated with faecal bile acids, lysozyme, IgA, butyl butyrate, and several activity-associated bacterial taxa, including *Turicibacter*, *Clostridium_* AP_143938, *Enterococcus_H_360604*, *Akkermansia*, and *Roseburia_A_166204*. Likewise, disease phenotype remained independently associated with *Clostridium_R_135822*, *Cavicella*, and *Blautia_A_141780*, whereas taxa displaying smaller effect sizes in the primary differential abundance analyses were no longer independently associated after adjustment for the main clinical covariates. To further investigate relationships among metabolites, association network analysis was performed (Figure S2). The resulting network identified coordinated associations among alcohols, ketones, SCFAs, and sulphur-containing compounds despite the overall conservation of global metabolomic composition.

**Figure 4:**
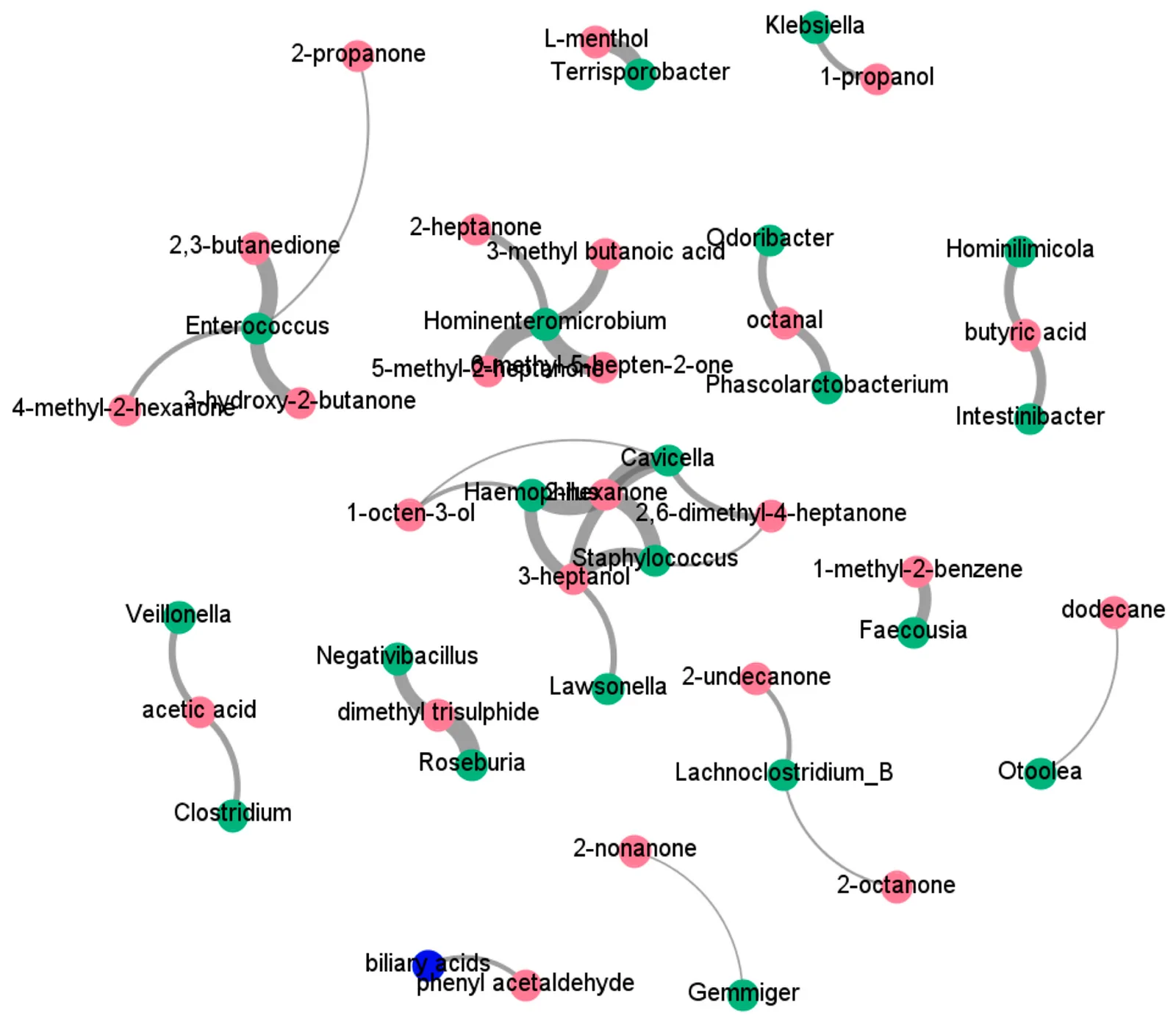
Integrated microbiota–metabolite network reveals coordinated luminal metabolic organization. Correlation network integrating bacterial genera, volatile organic compounds (VOCs), and bile acids. Nodes represent bacterial taxa (green), VOCs (pink), and markers (blue). Edges indicate significant positive correlations (ρ ≥ 0.70). Network topology demonstrates modular organization of microbial metabolism, identifying coordinated fermentation, lipid metabolism, and inflammation-associated metabolic modules within the luminal ecosystem.

To explore functional relationships among microbial taxa, metabolites, and host-derived biomarkers, an integrated association network was constructed (Figure 4).

The resulting network identified coordinated associations linking bacterial taxa, microbial metabolites, and host-derived biomarkers. A peripheral association linked bile acids with phenylacetaldehyde (ρ = 0.743), suggesting selective metabolic signalling between host bile metabolism and microbial aromatic amino-acid degradation.

*Enterococcus* was connected with several fermentation-derived ketones, including 2,3-butanedione (ρ = 0.909), 3-hydroxy-2-butanone (ρ = 0.881), 2-propanone (ρ = 0.708), and 4-methyl-2-hexanone (ρ = 0.752). The largest interaction hub involved multiple alcohols and medium-chain ketones (i.e., 2-hexanone (ρ up to 0.915), 3-heptanol (ρ up to 0.887), and 1-octen-3-ol (ρ = 0.735), 2,6-dimethyl-4-heptanone (ρ = 0.772), simultaneously correlated with several bacterial taxa, including *Cavicella*, *Staphylococcus*, *Haemophilus*, and *Lawsonella*. A conserved SCFA module linked classical anaerobic fermenters with acetic (e.g., *Clostridium,* ρ = 0.763 and *Veillonella*, ρ = 0.777) and butyric acid (e.g., *Intestinibacter,* ρ = 0.815 and *Hominilimicola*, ρ = 0.812), indicating partial preservation of carbohydrate fermentation pathways.

Additional modules highlighted amino-acid catabolism centred on *Hominenteromicrobium* (e.g., 6-methyl-5-hepten-2-one, ρ = 0.947, 5-methyl-2-heptanone, ρ = 0.940, 2-heptanone, ρ = 0.798, 3-methylbutanoic acid, ρ = 0.847) and a sulphur metabolic axis linking dimethyl trisulfide with *Roseburia* (ρ = 0.970) and *Negativibacillus* (ρ = 0.868).

Overall, the integrated network revealed coordinated relationships among microbial taxa, metabolites and host-derived biomarkers despite the relatively stable global composition of the luminal microbiota.

### 3.4. Mucosal Bacterial Microbiota Shows Phenotype-Associated Ecological Remodelling

**Figure 5:**
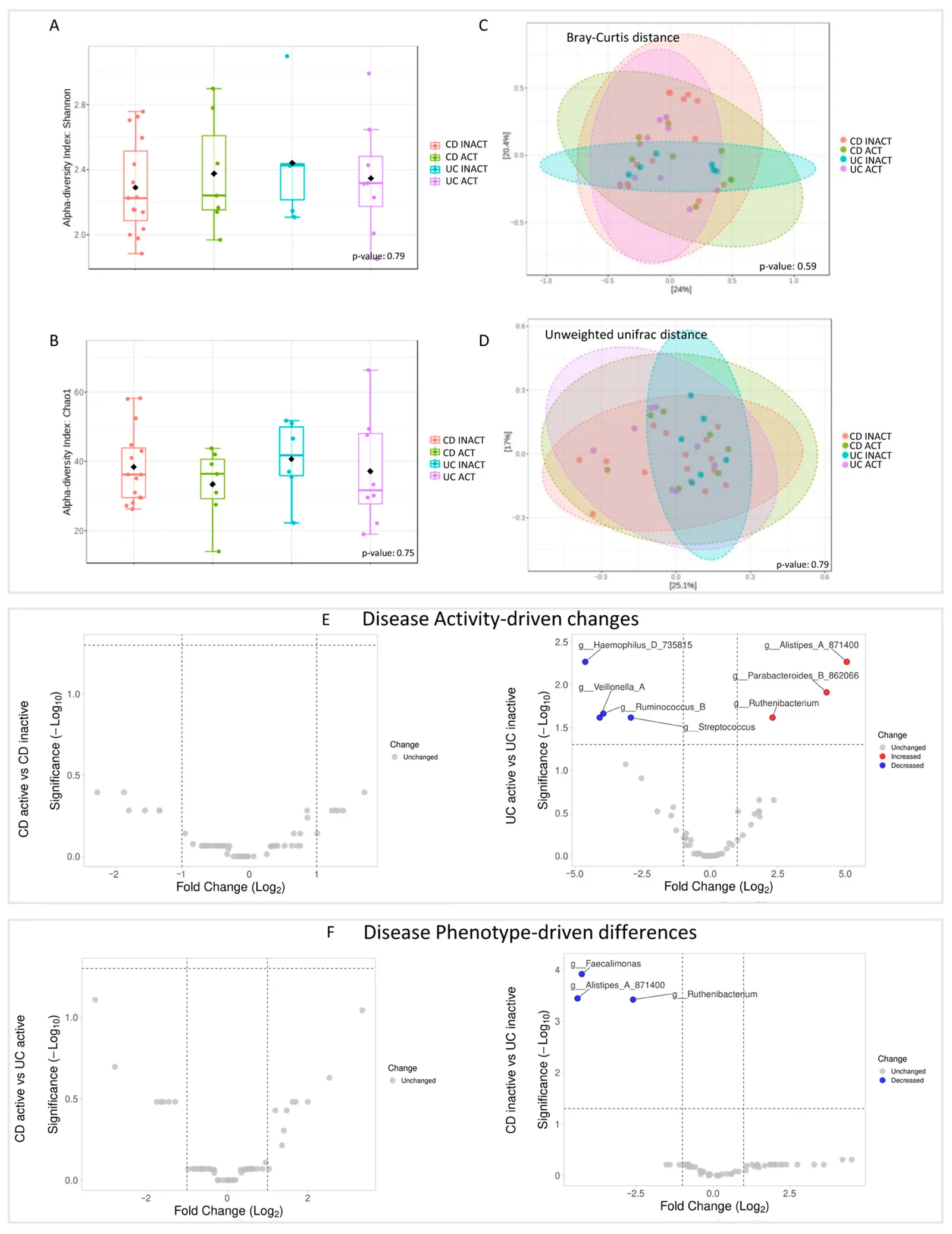
Mucosal bacterial microbiota displays phenotype-associated ecological remodelling. (**A**,**B**) Shannon and Chao1 alpha diversity indices across CD inactive, CD active, UC inactive, and UC active groups showing no significant differences (*p* = 0.79 and *p* = 0.75). Black diamonds indicate the group mean. (**C**,**D**) PCoA analyses based on Bray–Curtis and unweighted UniFrac distances showing absence of global phylogenetic clustering (*p* = 0.59 and *p* = 0.79). (**E**) Volcano plots of disease activity-associated taxa. (**F**) Volcano plots of phenotype-associated differences between CD and UC. Dashed lines indicate statistical significance thresholds.

To evaluate whether disease phenotype was associated with global changes in mucosal community diversity or structure, alpha and beta diversity analyses were performed. No significant differences were observed across disease groups, indicating preservation of the overall mucosal bacterial community architecture (Figure 5A–D).

To identify taxa specifically associated with disease activity or phenotype beyond global community structure, differential abundance analysis was subsequently performed. This analysis showed minimal changes in CD, whereas UC displayed marked activity-associated remodelling. Active UC was characterized by enrichment of *Alistipes*_A_871400, *Parabacteroides*_B_862066, *Ruthenibacterium*, and decreased abundance of *Haemophilus*_D_735815, *Veillonella*_A, *Ruminococcus*_B, and *Streptococcus*. No taxa differed between active CD and active UC, suggesting partially overlapping inflammatory signatures. During remission, CD showed reduced *Ruthenibacterium*, *Faecalimonas* and *Alistipes*_A_871400 compared with UC (Figure 5E,F).

Correlation analyses indicated distinct interaction patterns between active disease and remission, suggesting inflammation-associated restructuring of mucosal microbial communities (Figure S3). The resulting network consisted of 28 bacterial nodes connected by 24 significant correlations (ρ > 0.55), forming modular niche-specific communities.

### 3.5. Mucosal Fungal Community Defines Tissue-Specific Dysbiosis

**Figure 6:**
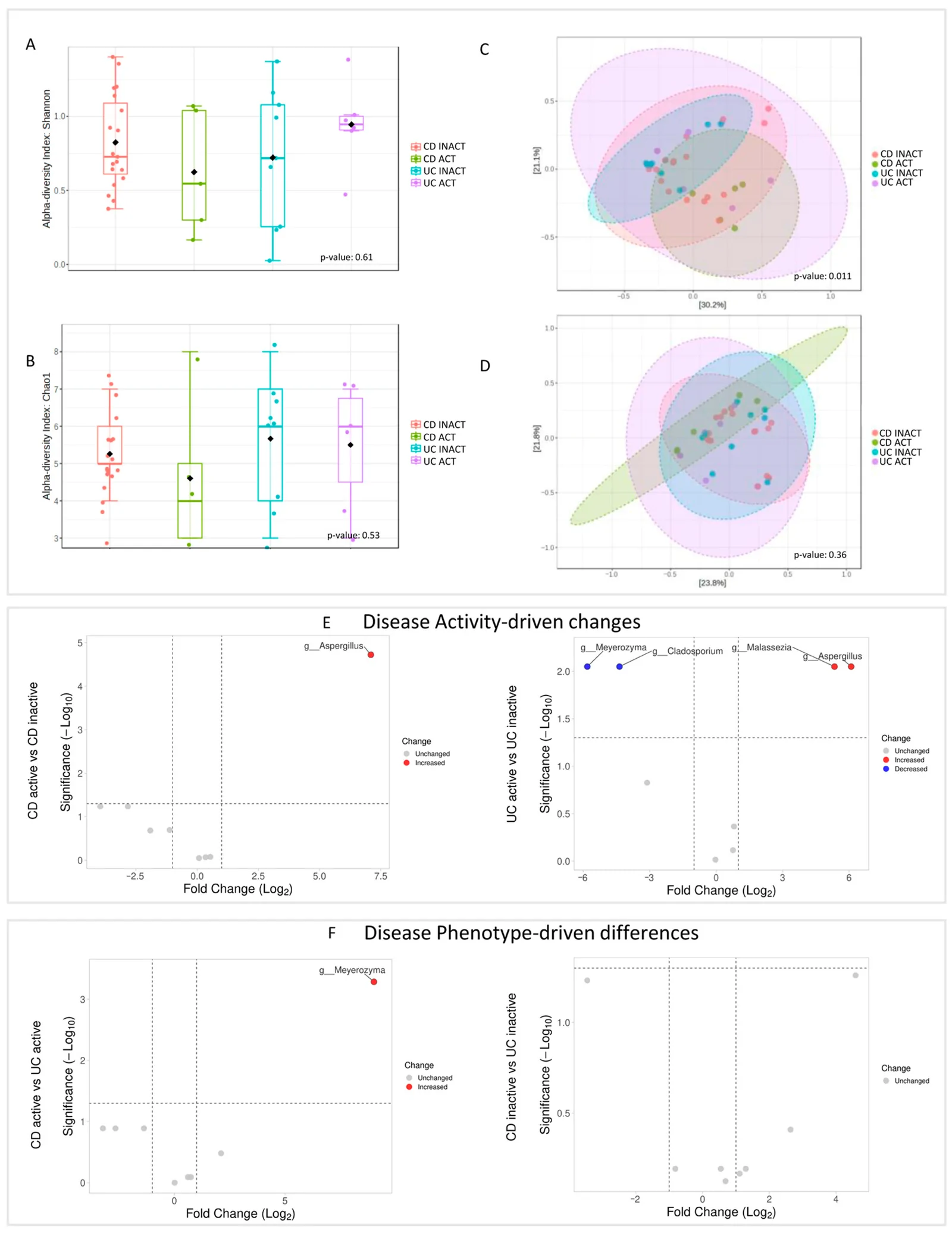
Mucosal mycobiota defines tissue-specific fungal dysbiosis in paediatric IBD. (**A**,**B**) Alpha diversity of mucosal fungal communities measured by Shannon and Chao1 indices across disease groups, showing no significant diversity differences (*p* = 0.61 and *p* = 0.53). Black diamonds indicate the group mean. (**C**,**D**) PCoA analyses based on Bray–Curtis and unweighted UniFrac distances showing significant community separation only for Bray–Curtis distance (*p* = 0.011), indicating compositional rather than phylogenetic restructuring. (**E**) Volcano plots illustrating activity-associated fungal taxa. (**F**) Volcano plots illustrating phenotype-associated fungal differences. Dashed lines indicate statistical significance thresholds.

To extend the ecological characterization of the mucosal compartment, we next investigated the tissue-associated fungal community. Fungal alpha diversity remained unchanged across groups (Shannon *p* = 0.61; Chao1 *p* = 0.53) (Figure 6A,B).

However, Bray–Curtis analysis demonstrated significant differences in community structure (*p* = 0.011), indicating activity-related compositional shifts not captured by diversity metrics (Figure 6C,D).

Active CD showed enrichment of *Aspergillus*. Active UC exhibited enrichment of *Aspergillus* and *Malassezia* together with reductions of *Meyerozyma* and *Cladosporium*. *Meyerozyma* was significantly higher in active CD compared with active UC, while inactive CD and UC showed no differences (Figure 6E,F).

Correlation analyses indicated altered fungal interaction patterns during active disease (Figure S4).

### 3.6. Cross-Kingdom Microbial Networks Reveal Mucosal Ecological Niches

**Figure 7:**
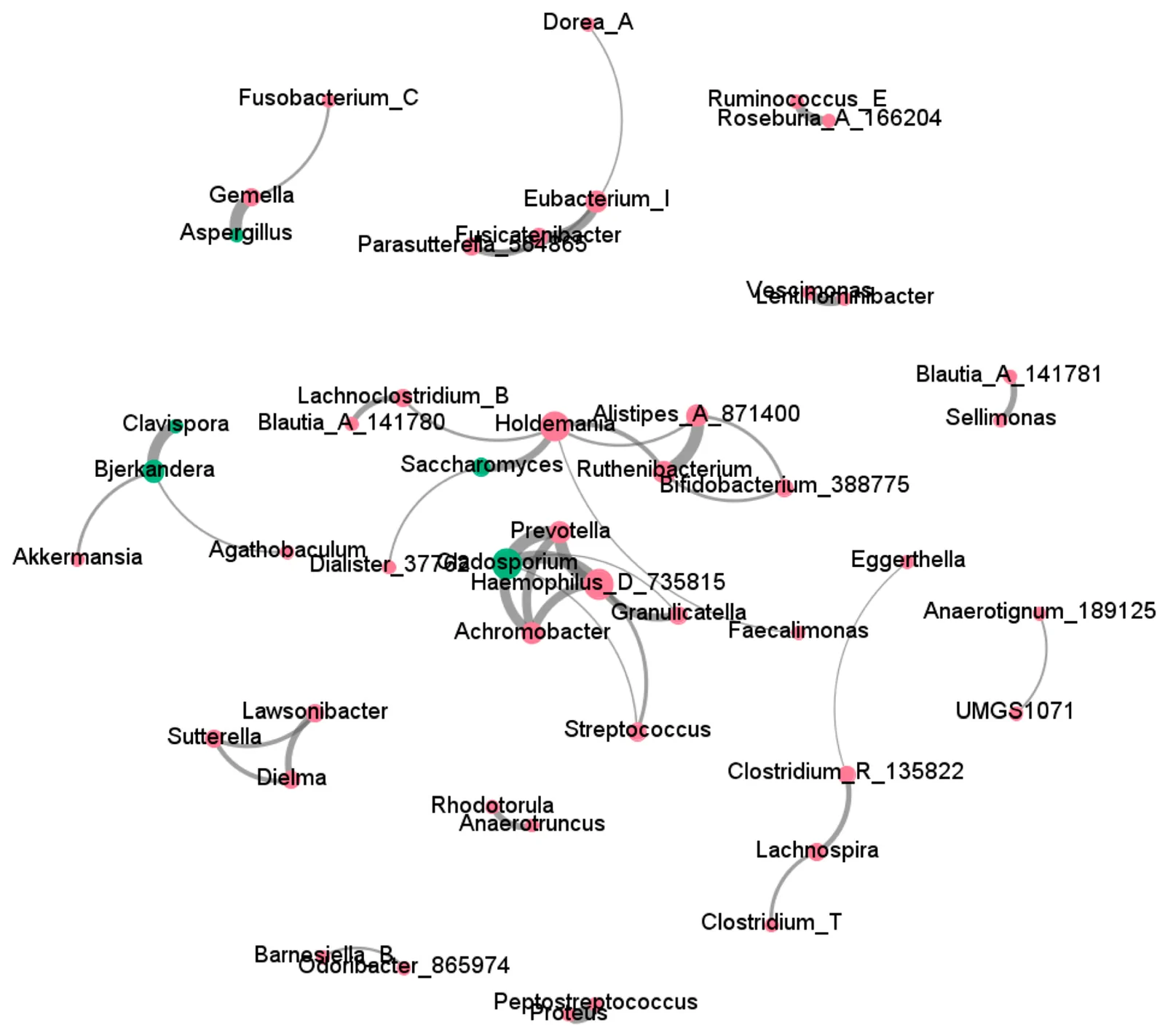
Cross-kingdom bacterial-fungal interaction network reveals structured mucosal ecological niches. Cross-kingdom correlation network derived from ileal biopsy samples integrating bacterial and fungal taxa. Nodes represent bacterial (pink) and fugal (green) taxa; edges indicate significant positive correlations (ρ ≥ 0.70), with edge thickness proportional to correlation strength. The network comprises ~52 nodes connected by 41 edges and displays a modular architecture including mucosal biofilm-like communities, anaerobic fermentation consortia, and inflammation-associated interaction modules.

Cross-kingdom analyses identified structured associations between bacterial and fungal taxa within the ileal mucosa (Figure 7).

Several fungi, including *Cladosporium* and *Aspergillus*, were associated with oral-associated, mucin-associated and fermentative bacterial taxa.

### 3.7. Integration Between Luminal and Mucosal Bacterial Communities Highlighted Compartment-Specific Ecological Patterns

**Figure 8:**
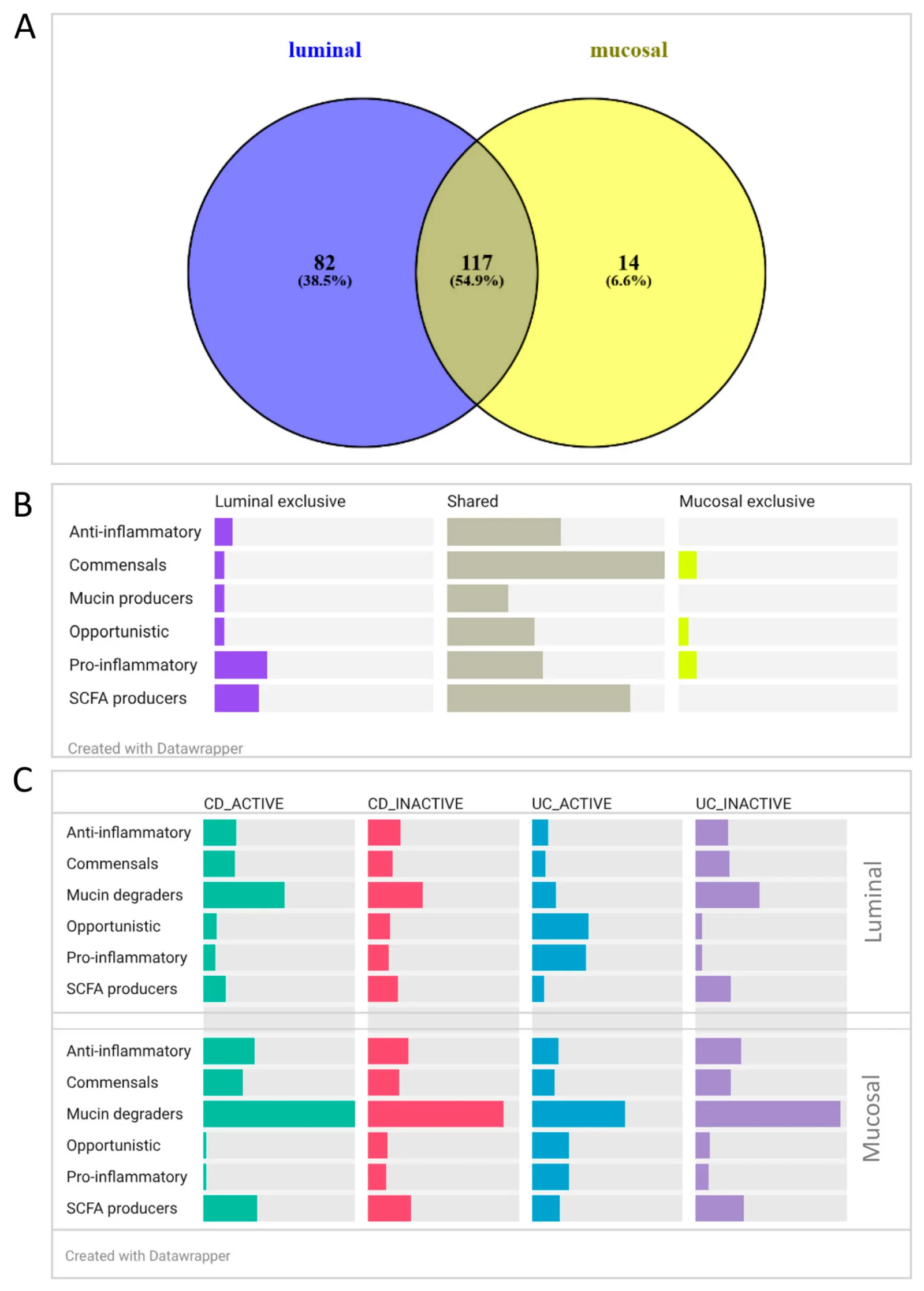
Spatial ecological compartmentalization between luminal and mucosal microbiota. (**A**) Venn diagram showing taxonomic overlap between faecal (luminal) and ileal mucosal bacterial communities, identifying compartment-exclusive and shared genera. (**B**) Stacked bar plots representing frequency of bacterial genera categorized according to reported ecological functions within luminal-exclusive, mucosal-exclusive, and shared fractions. (**C**) Relative abundance of functional bacterial categories stratified by disease status, including anti-inflammatory taxa, commensals, mucus-associated bacteria, opportunistic organisms, pro-inflammatory taxa, and short-chain fatty acid (SCFA) producers based on published functional annotations (Supplementary Table S4). These analyses illustrate spatial segregation and differential distribution of bacterial functional categories across intestinal compartments in paediatric IBD.

Finally, to define the spatial organization of the intestinal ecosystem, luminal, and mucosal bacterial communities were directly compared to identify shared and compartment-specific ecological features (Figure 8A).

Although 117 genera were shared (Jaccard similarity = 0.55; Sørensen index = 0.71), 89.3% of mucosal taxa were detectable in faeces, whereas only 58.8% of luminal taxa colonized the mucosa, suggesting selective enrichment of bacterial taxa at the epithelial interface.

Classification of bacterial taxa according to published ecological and functional annotations (Supplementary Table S4) revealed distinct distributions across intestinal compartments (Figure 8B). Luminal-exclusive taxa were enriched in SCFA producers and pro-inflammatory bacterial groups, whereas mucosal communities showed a higher representation of opportunistic and inflammation-associated bacteria. The shared fraction contained the majority of commensal taxa while also including opportunistic bacterial groups.

Stratification by disease activity showed that active UC was associated with reduction of mucin degraders and SCFA-producing bacterial groups together with an increased representation of pro-inflammatory taxa across both compartments, consistent with a coordinated disruption of luminal and mucosal ecosystem organization during active inflammation (Figure 8C).

Overall, these findings indicate that luminal and mucosal compartments provide complementary microbial information, with luminal communities more closely associated with inflammatory activity and mucosal communities retaining more stable phenotype-associated signatures.

## 4. Discussion

In this integrated multi-omics analysis, combining luminal and mucosal microbial communities with metabolomic profiles and host-derived biomarkers, our findings suggest that distinct intestinal compartments may provide complementary biological information on paediatric IBD. Rather than representing a homogeneous dysbiotic condition, paediatric IBD appears to involve compartment-specific host–microbiome interactions, with disease activity being more consistently associated with the luminal ecosystem, whereas phenotype-associated signatures were more evident in the mucosal compartment, particularly within the mycobiome. These findings support a systems-level model in which different biological layers contribute non-redundant insights into disease mechanisms. Given that both CD and UC are characterized by established dysbiosis relative to healthy individuals, the relatively modest global differences observed between the two diseases are not unexpected. Rather, disease-specific signatures emerged from the integration of compartment-specific microbial, metabolic, and host-derived features. Similarly, comparisons according to disease activity were performed between patients sharing the same chronic inflammatory disease, who were frequently receiving medical therapies known to influence the gut microbiota. Therefore, rather than expecting major shifts in overall community structure, identifying selective ecological signatures was considered to be more biologically informative.

Faecal and urinary biomarkers further supported this framework. Lysozyme, bile acids, and indican were primarily associated with inflammatory activity, particularly in UC, and their reciprocal correlations suggest coordinated interactions between innate immune responses and microbial metabolism during intestinal inflammation. Despite these associations, none correlated with conventional clinical activity indices, indicating that these biomarkers capture biological processes only partially reflected by clinical scoring systems. Elevated urinary indican further supports increased proteolytic metabolism during inflammation [64]. Recent evidence has shown that active IBD is associated with altered expression of ileal bile acid transporters, including the apical sodium-dependent bile acid transporter (ASBT), indicating that disturbances in bile acid homeostasis are multifactorial and cannot be inferred solely from total faecal bile acid measurements [65].

Collectively, these findings suggest that host-derived biomarkers are more closely associated with inflammatory burden than with disease phenotype, a finding that remained robust after adjustment for the main clinical covariates. Additional multivariable regression analyses adjusting for age, disease duration, biological therapy, 5-ASA treatment, disease phenotype, and disease activity further supported the robustness of these findings, indicating that the principal biomarker, metabolite, and microbial signatures were largely independent of the main clinical covariates.

Despite these inflammatory signals, the luminal bacterial community largely preserved its overall structure, indicating that paediatric IBD is characterized more by functional remodelling than by widespread disruption of community composition. Inflammatory activity was associated with selective alterations affecting metabolically relevant taxa. Active CD showed depletion of several butyrate-producing bacteria together with enrichment of mucus-associated and succinate-related taxa, suggesting reduced anti-inflammatory metabolic potential and adaptation to altered substrate availability within the inflamed intestine. In particular, expansion of *Akkermansia* may reflect increased mucus turnover and epithelial stress rather than an exclusively beneficial role [66], whereas enrichment of propionate and succinate-associated microorganisms is consistent with reorganization of microbial cross-feeding under inflammatory conditions [67,68,69].

Changes in UC were more restricted and mainly consisted of depletion of butyrate-producing taxa together with expansion of inflammation-adapted microorganisms, consistent with impaired epithelial support and barrier function [70,71]. Direct comparison between active CD and UC further highlighted phenotype-associated differences, with CD showing enrichment of anaerobic fermentative taxa and UC displaying increased abundance of aerotolerant and oral-associated bacteria. This pattern is consistent with inflammation-associated epithelial oxygenation favouring facultative anaerobes at the expense of obligate anaerobic commensals [72,73,74].

Importantly, several microbial signatures persisted during remission. Patients with CD maintained depletion of butyrate-producing taxa, suggesting incomplete ecological recovery despite clinical quiescence. Similar persistence of inflammation-independent microbial alteration during CD remission has recently been reported [75]. Our findings extend these observations by showing that luminal and mucosal host–microbiome interactions provide complementary information on disease activity and phenotype. Network analyses further indicated that inflammatory activity remodels microbial interactions more extensively than overall community composition. Rather than diffuse dysbiosis, microbial communities retained structured interaction patterns, suggesting preservation of cooperative fermentative networks despite taxonomic rearrangements.

A similar picture emerged from metabolomic profiling. Although, global metabolomic composition largely overlapped across groups, targeted analyses identified clear activity-associated metabolic remodelling. Active UC showed enrichment of several alcohols and ketones previously associated with intestinal inflammation [26,30,76,77,78], whereas metabolites with reported anti-inflammatory properties, including beta-caryophyllene and 1-propanol were reduced during active disease [79,80]. Metabolite network analysis further revealed highly interconnected functional modules centred on a limited number of hub metabolites, supporting coordinated metabolic reprogramming rather than widespread disruption of the metabolome.

Integrated microbiome–metabolome analyses further supported this functional interpretation. *Enterococcus* emerged as a central hub associated with fermentation-derived ketones, suggesting increased integration of aerotolerant microorganisms within inflammation-associated metabolic networks [72]. At the same time, persistent associations between SCFAs and classical anaerobic fermenters, indicate partial resilience of core carbohydrate fermentation pathways despite ecological stress [81,82]. Additional modules involving amino-acid and sulphur metabolism further suggest enhanced proteolysis and redox adaptation during epithelial injury [83]. Together, these findings suggest that inflammatory activity is associated with functional remodelling of host–microbiome interactions rather than widespread disruption of community structure [14].

In contrast, phenotype-associated ecological differences were more evident within the mucosal compartment than in the luminal ecosystem. While bacterial differences were modest, the fungal community displayed clearer phenotype-associated compositional changes, supporting the complementary contribution of the mucosal compartment to disease characterization. The standardized analysis of the ileal mucosal compartment also provided the opportunity to investigate bacterial and fungal communities within the same ecological niche. Although UC primarily affects the colonic mucosa, accumulating evidence indicates that the terminal ileum is not biologically quiescent, even in the absence of macroscopic inflammation, but undergoes transcriptomic, immune, and microbial alterations associated with the disease [84,85,86]. Furthermore, growing evidence indicates that fungi actively contribute to IBD pathogenesis not only through direct modulation of host immune responses but also by shaping bacterial community structure and function through cross-kingdom interactions [10,87,88]. Rather than acting as independent microbial kingdoms, bacteria and fungi constitute an interconnected intestinal ecosystem whose ecological balance is increasingly recognized as a key determinant of intestinal homeostasis and inflammation [87]. Nevertheless, studies investigating terminal ileal biopsies have almost exclusively focused on bacterial communities, whereas characterization of the ileal mucosal mycobiome in UC remains virtually unexplored. By integrating mucosal bacteriome and mycobiome profiles with luminal microbiota, metabolomics, and host-derived biomarkers, our study provides an ecosystem-level perspective of host–microbiome interactions that extends beyond conventional bacteria-centred approaches. Active UC was characterized by enrichment mucus-adapted bacteria capable of exploiting host-derived substrates released during epithelial injury [89], together with depletion of taxa involved in cooperative fermentative interactions and maintenance of mucosal metabolic homeostasis [90]. Unlike the lumen, the mucosal compartment also showed distinct responses among aerotolerant genera such as *Streptococcus* and *Haemophilus* [29], further supporting the concept that luminal and mucosal niches provide complementary information regarding disease biology.

Our data further identify the mucosal mycobiota as an additional component of host-associated microbial communities. *Aspergillus* emerged as a consistent marker of inflammatory activity in both CD and UC [91], whereas active UC was additionally characterized by enrichment of *Malassezia* and depletion of *Meyerozyma* and *Cladosporium*. Conversely, the higher abundance of *Meyerozyma* in active CD compared with active UC supports previous observations suggesting a potentially protective role for this genus.

These findings are consistent with previous reports from our group. Paediatric UC has been associated with enrichment of inflammatory fungal genera, including *Cladosporium* and *Aureobasidium* [19], whereas adult UC cohorts consistently showed increased *Malassezia* abundance [30], supporting its proposed pro-inflammatory role [92]. In contrast, studies in primary sclerosing cholangitis-associated UC reported reduced abundance of both *Meyerozyma* and *Malassezia*, further supporting a potentially beneficial role for *Meyerozyma* [40,93]. Together, these observations further support a potential contribution of fungal communities in host–microbiome interactions and represent a potentially important, yet often overlooked, component of paediatric IBD biology. Cross-kingdom analyses further suggest that bacterial and fungal populations are interconnected components of the intestinal ecosystem. The coexistence of oral-associated, mucin-associated, and commensal anaerobic microorganisms suggests coordinated ecological responses to inflammation and highlights the potential contribution of fungal communities to microbial community stability and host interactions.

Integration of luminal and mucosal datasets ultimately revealed limited taxonomic overlap between the two niches. The mucosa represented a selective environment enriched in tissue-associated and inflammation-adapted microorganisms, whereas the lumen remained relatively enriched in SCFA-producing bacteria characteristic of homeostatic communities. During active UC, coordinated depletion of mucin degraders and SCFA producers together with expansion of inflammation-adapted taxa across both compartments suggests that inflammatory pressure progressively reshapes microbial organization through altered oxygen availability, mucus turnover, and host-derived nutrient availability [94].

Overall, our findings support the concept that luminal and mucosal host–microbiome interactions provide complementary information on inflammatory activity and disease phenotype. Integrating bacterial and fungal communities with metabolomic profiles and host-derived biomarkers moves beyond descriptive microbiome profiling and may provide a biologically informed framework for investigating disease mechanisms and identifying clinically relevant microbial signatures [94].

This study has several limitations. Its cross-sectional design precludes assessment of temporal microbiome dynamics and causal relationships, while the moderate cohort size and heterogeneous therapeutic regimens may have influenced microbial composition. Differences in biologic therapy and 5-ASA use reflect routine clinical management of CD and UC and may therefore have contributed to part of the observed microbial variability. Although multivariable regression analyses were performed to account for the main clinical confounders, the limited sample size may have reduced the power to detect independent associations for variables with small effect sizes. Finally, because mucosal biopsies were systematically obtained from the terminal ileum, the findings in UC should be interpreted as reflecting disease-associated alterations of the ileal mucosal ecosystem rather than the primary colonic site of inflammation. Additionally, detailed dietary information was unavailable, although the relatively homogeneous Mediterranean dietary habits of the study population likely reduced dietary variability. Future longitudinal studies in larger and clinically balanced cohorts will be important to determine the stability and prognostic relevance of the biological signatures identified here.

## 5. Conclusions

This study suggests that integrating luminal and mucosal bacterial and fungal communities with metabolomic profiles and host-derived biomarkers provides complementary insights into paediatric IBD biology. Disease activity was more consistently associated with the luminal ecosystem through dynamic immune–metabolic interactions, whereas phenotype-associated signatures were more evident in the mucosal compartment, particularly within the mycobiome. Together, these findings support the concept that integrated multi-omic analyses of distinct intestinal compartments can provide complementary information on host–microbiome interactions in paediatric IBD and may facilitate the identification of biologically meaningful microbial signatures.

## Figures and Tables

**Table 1:** Clinical and demographic characteristics of the study population according to disease type.

Variable	UC (n = 24)	CD (n = 22)	*p*-Value
Age (years) (mean ± SD)	11.9 ± 4.4	14.8 ± 3.1	0.01
BMI ^1^ (kg/m^2^)	18.7 ± 3.6	19.6 ± 2.2	0.30
Disease duration (years)	1.8 ± 2.9	3.9 ± 4.0	0.04
PCDAI ^2^/PUCAI ^3^	10.4 ± 17.4	8.5 ± 10.6	0.68
SES-CD ^4^/Matts score	7.8 ± 8.5	5.2 ± 4.3	0.18
Gender			
Male N (%)	10 (41.7%)	14 (63.6%)	0.16
Female	14 (58.3%)	8 (36.4%)	
Disease activity			
Active disease	10 (41.7%)	6 (27.3%)	0.30
Inactive disease	14 (58.3%)	16 (72.7%)	
Biological therapy			
Yes	3 (12.5%)	10 (45.5%)	0.01
No	21 (87.5%)	12 (54.5%)	
Immunosuppressive therapy			
Yes	3 (12.5%)	7 (31.8%)	0.11
No	21 (87.5%)	15 (68.2%)	
^5^ 5-ASA therapy			
Yes	12 (50.0%)	5 (22.7%)	0.04
No	12 (50.0%)	17 (77.3%)	
Disease localization			
Distal colonic disease	16 (66.7%)	4 (18.2%)	<0.001
Extensive colonic disease	8 (33.3%)	1 (4.5%)	
Ileal involvement	0	17 (77.3%)	

^1^ BMI, body mass index; ^2^ PCDAI, Paediatric Crohn’s Disease Activity Index; ^3^ PUCAI, Paediatric Ulcerative Colitis Activity Index; ^4^ SES-CD, Simple Endoscopic Score for Crohn’s Disease, ^5^ 5-ASA, 5-aminosalicylic acid.

**Table 2:** Linear regression analysis of faecal and urinary biomarkers.

Dependent	Predictor	β (Slope)	95% CI	*p*-Value	R^2^	Variance Explained
Lysozyme	IgA	0.302	0.129–0.475	0.001	0.298	29.8%
Indican	Zpn	0.208	0.038–0.378	0.018	0.177	17.7%
Bile acids	Lysozyme	0.844	0.100–1.587	0.028	0.21	21.0%
Lysozyme	Indican	−0.463	−1.012–0.085	0.094	0.093	9.3%
Bile acids	IgA	0.351	−0.066–0.769	0.095	0.127	12.7%
IgA	Indican	−0.561	−1.535–0.412	0.248	0.046	4.6%
IgA	Zpn	0.193	−0.290–0.676	0.421	0.022	2.2%
Bile acids	Indican	−0.285	−1.388–0.817	0.595	0.014	1.4%
Lysozyme	Zpn	0.064	−0.205–0.333	0.631	0.008	0.8%
Bile acids	Zpn	0.064	−0.528–0.655	0.825	0.002	0.2%

## Data Availability

The datasets generated and analysed in this study are available from the corresponding author upon reasonable request, in accordance with ethical and privacy regulations.

## Supplementary Materials

The following supporting information can be downloaded at: https://www.mdpi.com/article/10.3390/microorganisms14092027/s1, Figure S1. Fecal bacterial co-occurrence network. Co-occurrence network analysis illustrating bacterial ecological interactions within fecal samples across disease phenotypes (CD active, CD inactive, UC active, UC inactive). Nodes represent bacterial taxa, and edges indicate significant correlations between taxa (red edges denote positive correlations), reflecting potential ecological associations within the luminal microbiota. A Firmicutes-dominated cluster, including *Hominibacterium*, *Anaeromassilibacillus*, *Bilophila*, and *Hominicoprocola*, was enriched in UC inactive, suggesting ecological stability during remission. A second cluster composed of SCFA-related taxa (i.e., *Merdimonas*, *Phascolarctobacterium*, *Odoribacter*, *Alistipes*, and *Parabacteroides)* predominated in CD active samples. A mixed transitional module included *Dysosmobacter*, *Gordonibacter*, *Clostridium*, and *Ruthenibacterium,* reflecting shared ecological interactions across disease states. UC activity displayed distinct low interconnected networks, enriched in oral-associated and inflammation-adapted taxa (i.e., *Bulleidia*, *Sarcina*, *Haemophilus*, *Cavicella*, *Dielma* and *Sellimonas),* suggesting a disease-specific microbial signature.; Figure S2. Fecal metabolomic association network. Network analysis of fecal metabolomic profiles showing relationships among detected metabolites. Nodes represent individual metabolites, whereas edges indicate significant pairwise associations. Red edges denote positive correlations and blue edges denote negative correlations; edge thickness reflects correlation strength. Green highlighted nodes identify highly connected metabolic hubs within the network. Highly connected hubs included 4-methylphenol (degree = 8; betweenness = 326.92), p-cymene (degree = 7; betweenness = 300.95) and 3-methylbutanoic acid (degree = 7; betweenness = 244.90), indicating central roles in coordinating gut metabolic interactions. Additional nodes with high betweenness centrality included 1-hexanol (283.27), 5-methyl-3-hexanone (274.53), 4-methyl-2-hexanone (242.70), and acetic acid (216.11), suggesting that these compounds may act as key intermediates linking different metabolic clusters.; Figure S3. Mucosal bacterial co-occurrence network. Co-occurrence network analysis depicting bacterial ecological interactions within ileal mucosal biopsy samples across disease phenotypes (CD active, CD inactive, UC active, UC inactive). Nodes represent mucosa-associated bacterial taxa, and edges indicate significant correlations (red edges denote positive correlations), revealing phenotype-associated ecological organization at the epithelial interface. Active disease modules included aerotolerant and opportunistic taxa centered on *Escherichia* and *Enterococcus*, consistent with oxygen-adapted inflammatory microbiota. In contrast, UC inactive samples retained anaerobic consortia including *Ruthenibacterium* and *Alistipes*, suggesting ecological stabilization during remission.; Figure S4. Mucosal fungal co-occurrence network. Co-occurrence network analysis illustrating fungal ecological interactions within ileal mucosal biopsy samples across disease phenotypes (CD active, CD inactive, UC active, UC inactive). Nodes correspond to fungal taxa and edges represent significant correlations (blu edges denote positive correlations), highlighting tissue-specific mycobiome ecological structures.; Supplementary Table S1. Summary of sequencing quality control metrics across the three sequencing datasets. Raw reads and high-quality reads after filtering are reported as median (IQR). ASVs retained indicate the total number of amplicon sequence variants retained after sequence processing and filtering; Supplementary Table S2. Mean ± standard deviation (SD) values of biomarkers across the four clinical groups (UC inactive, UC active, CD inactive, and CD active); Supplementary Table S3. Multivariable linear regression analyses evaluating the independent associations of age, disease duration, biological therapy, 5-ASA treatment, disease activity, and disease phenotype with the principal host-derived biomarkers, faecal metabolites, and representative bacterial taxa. Values represent *p*-values for each independent variable in the multivariable model. Statistically significant associations (*p* < 0.05) are highlighted in bold; Supplementary Table S4. Functional classification of bacterial genera included in the ecological analyses. Bacterial genera were assigned to functional categories based on manually curated evidence from published experimental studies and authoritative review articles. Because the ecological role of several genera is species- and context-dependent, multiple functional categories were assigned when supported by the literature. Representative references supporting ezqach functional annotation are provided.
