# Supplementary material for: Integrated Multi-Omics Reveals Complementary Luminal and Mucosal Host–Microbiome Interactions Associated with Disease Activity and Phenotype in Paediatric Inflammatory Bowel Disease
Source: Microorganisms. 2026 Sep 11;14(9):2027. doi: 10.3390/microorganisms14092027 (PMC13609212; doi:10.3390/microorganisms14092027)
Supplement: Supplementary file 1 [file microorganisms-14-02027-s001.zip › microorganisms-4461335-supplementary.pdf]

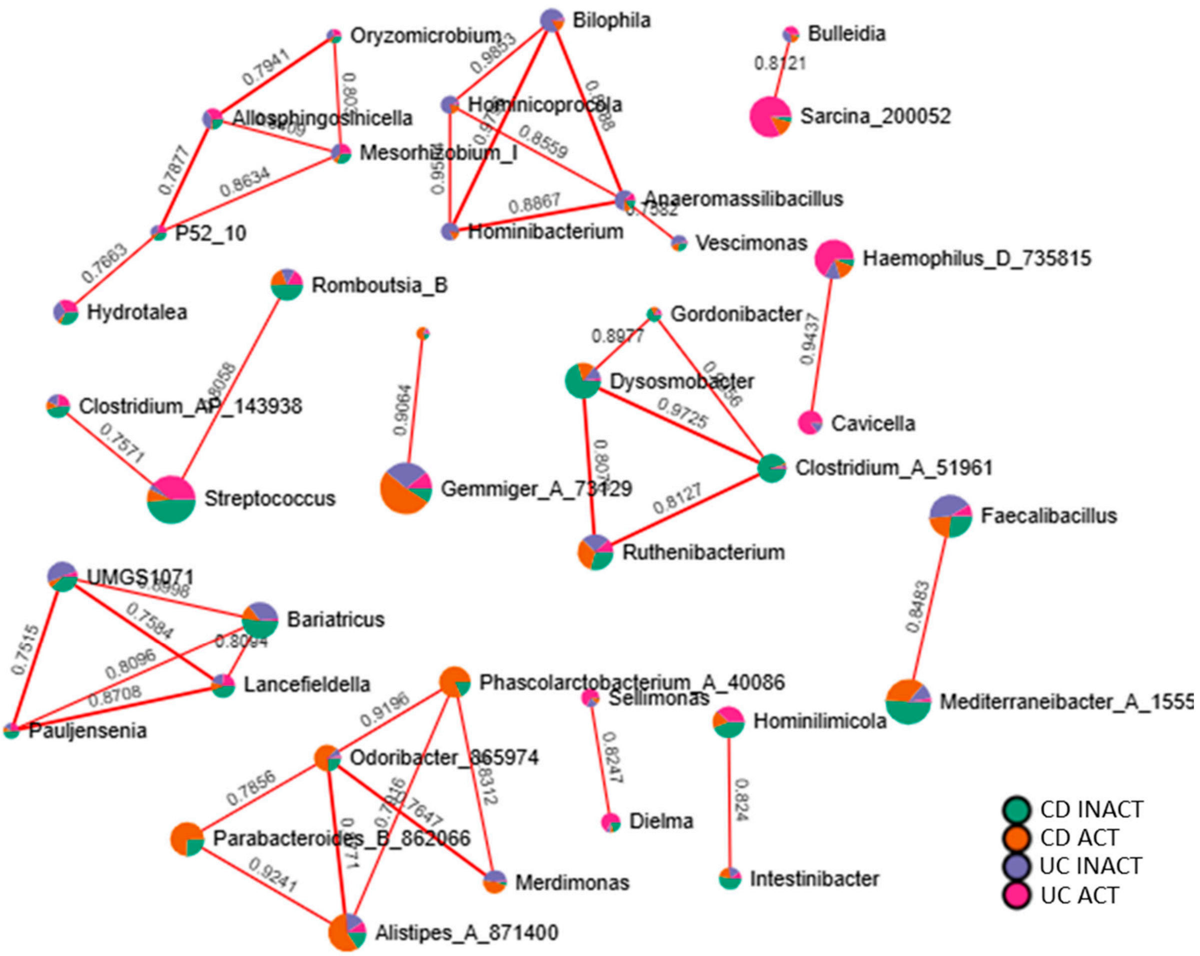

Figure S1. Fecal bacterial co-occurrence network. Co-occurrence network analysis illustrating bacterial ecological interactions within fecal samples across disease phenotypes (CD active, CD inactive, UC active, UC inactive). Nodes represent bacterial taxa, and edges indicate significant correlations between taxa (red edges denote positive correlations), reflecting potential ecological associations within the luminal microbiota. A Firmicutes-dominated cluster, including *Hominibacterium*, *Anaeromassilibacillus*, *Bilophila*, and *Hominicoprocola*, was enriched in UC inactive, suggesting ecological stability during remission. A second cluster composed of SCFA-related taxa (i.e., *Merdimonas*, *Phascolarctobacterium*, *Odoribacter*, *Alistipes*, and *Parabacteroides)* predominated in CD active samples. A mixed transitional module included *Dysosmobacter*, *Gordonibacter*, *Clostridium*, and *Ruthenibacterium,* reflecting shared ecological interactions across disease states. UC activity displayed distinct low interconnected networks, enriched in oral-associated and inflammation-adapted taxa (i.e., *Bulleidia*, *Sarcina*, *Haemophilus*, *Cavicella*, *Dielma* and *Sellimonas),* suggesting a disease-specific microbial signature.

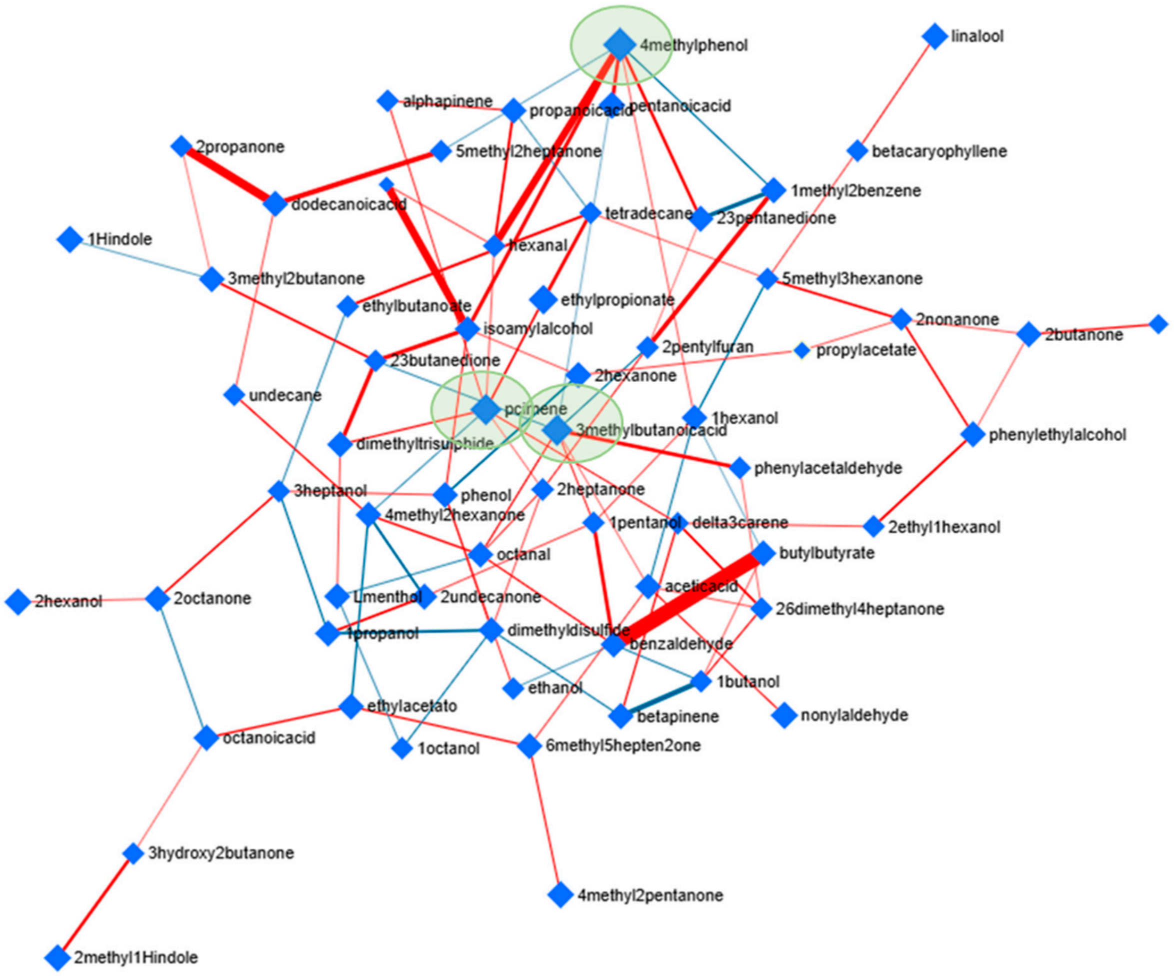

Figure S2. Fecal metabolomic association network. Network analysis of fecal metabolomic profiles showing relationships among detected metabolites. Nodes represent individual metabolites, whereas edges indicate significant pairwise associations. Red edges denote positive correlations and blue edges denote negative correlations; edge thickness reflects correlation strength. Green highlighted nodes identify highly connected metabolic hubs within the network. Highly connected hubs included 4-methylphenol (degree = 8; betweenness = 326.92), p-cymene (degree = 7; betweenness = 300.95) and 3-methylbutanoic acid (degree = 7; betweenness = 244.90), indicating central roles in coordinating gut metabolic interactions. Additional nodes with high betweenness centrality included 1-hexanol (283.27), 5-methyl-3-hexanone (274.53), 4-methyl-2-hexanone (242.70), and acetic acid (216.11), suggesting that these compounds may act as key intermediates linking different metabolic clusters.

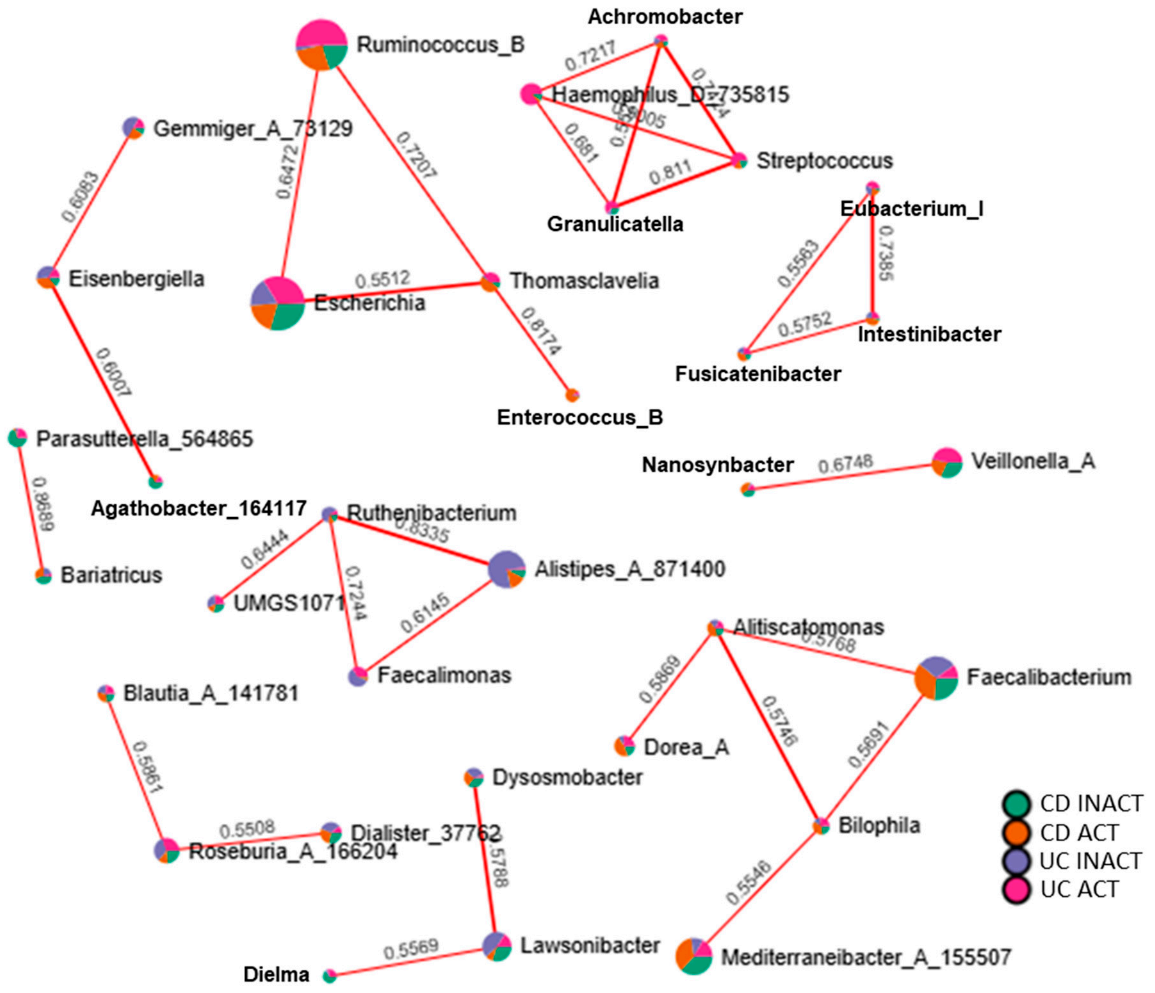

Figure S3. Mucosal bacterial co-occurrence network. Co-occurrence network analysis depicting bacterial ecological interactions within ileal mucosal biopsy samples across disease phenotypes (CD active, CD inactive, UC active, UC inactive). Nodes represent mucosa-associated bacterial taxa, and edges indicate significant correlations (red edges denote positive correlations), revealing phenotype-associated ecological organization at the epithelial interface. Active disease modules included aerotolerant and opportunistic taxa centered on *Escherichia* and *Enterococcus*, consistent with oxygen-adapted inflammatory microbiota. In contrast, UC inactive samples retained anaerobic consortia including *Ruthenibacterium* and *Alistipes*, suggesting ecological stabilization during remission.

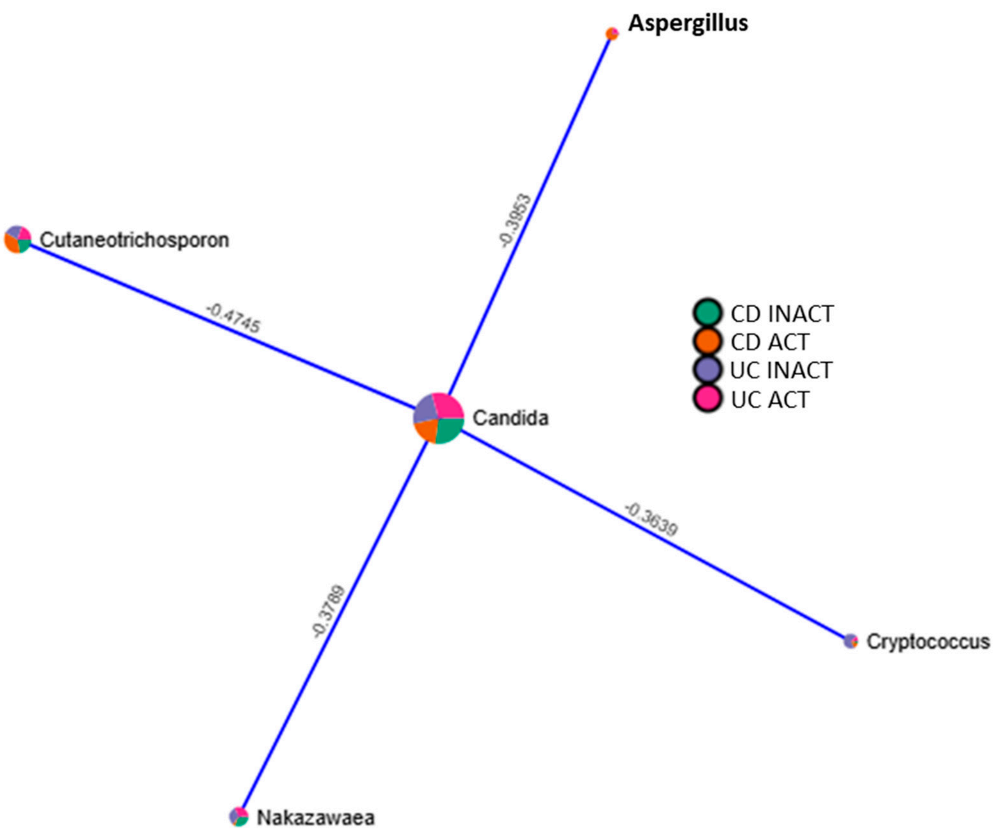

Figure S4. Mucosal fungal co-occurrence network. Co-occurrence network analysis illustrating fungal ecological interactions within ileal mucosal biopsy samples across disease phenotypes (CD active, CD inactive, UC active, UC inactive). Nodes correspond to fungal taxa and edges represent significant correlations (blu edges denote positive correlations), highlighting tissue-specific mycobiome ecological structures.

Supplementary Table S1. Summary of sequencing quality control metrics across the three sequencing datasets. Raw reads and high-quality reads after filtering are reported as median (IQR). ASVs retained indicate the total number of amplicon sequence variants retained after sequence processing and filtering.

Dataset	Samples (n)	Raw reads (median, IQR)	High-quality reads after filtering (median, IQR)	ASVs retained
Faecal bacteriome (16S)	35	120,895 (75,443–186,112)	109,334 (69,130–171,583)	2762
Mucosal bacteriome (16S)	38	67,344 (29,139–146,181)	67,173 (29,115–145,951)	1707
Mucosal mycobiome (ITS2)	39	32,054 (24,430–42,948)	26,531 (21,385–36,150)	174

Supplementary Table S2. Mean ± standard deviation (SD) values of biomarkers across the four clinical groups (UC inactive, UC active, CD inactive, and CD active).

Markers (average ± SD)	CD_INACT	CD_ACT	UC_INACT	UC_ACT
Bile acids (mg/g feces)	8.82 ± 9.84	2.66 ± 2.45	21.25 ± 14.36	1.21 ± 1.93
Lysozyme (µmol/g feces)	1848.59 ± 1511.08	1482.85 ± 788.55	2691.39 ± 2096.02	1341.12 ± 443.07
Zpn (ng/ml)	181.64 ± 146.75	98.05 ± 54.49	301.80 ± 196.23	203.27 ± 147.37
IgA (µg/ml)	3248.12 ± 2406.57	2052.67 ± 1126.15	5101.45 ± 3265.66	2158.67 ± 1678.76
Indicane (mg/dL)	13.86 ± 5.35	7.30 ± 1.94	10.53 ± 3.76	11.98 ± 8.62

Supplementary Table S3. Multivariable linear regression analyses evaluating the independent associations of age, disease duration, biological therapy, 5-ASA treatment, disease activity and disease phenotype with the principal host-derived biomarkers, faecal metabolites and representative bacterial taxa. Values represent p-values for each independent variable in the multivariable model. Statistically significant associations ($p < 0.05$) are highlighted in bold.

**linear regression (p value)**	**Age**	**Disease duration**	**Biological treatment**	**5-AZA treatment**	**Disease activity**	**Disease phenotype**
bile acids	0.6348	0.9719	0.7295	0.5215	**0.0023**	0.1227
lysozyme	0.7686	0.3809	0.3748	0.8103	**0.0020**	0.8268
indican	0.8714	0.8284	0.7299	0.8162	0.3087	0.9630
IgA	0.6174	0.6562	0.0604	0.4752	**0.0325**	0.8848
Zpn	0.5280	0.9292	0.9496	0.4638	0.1760	0.0557
butylbutyrate	0.3389	0.7250	0.5530	0.4589	**0.0221**	0.0609
aceticacid	0.1055	0.4447	0.5587	0.1207	0.1302	0.8820
phenylethylalcohol	0.6101	0.4144	0.3968	0.6452	0.6167	0.1492
3hydroxy2butanone	0.8109	0.7502	0.9442	0.7000	0.1260	0.2692
1octen3ol	0.6093	0.9380	0.7041	0.8486	0.5111	0.1177
Turicibacter	0.8377	0.6530	0.3822	0.4177	**0.0050**	0.2644
*Clostridium*_AP_143938	0.6409	0.0638	0.6258	0.3501	**0.0035**	0.4529
*Enterococcus*_H_360604	0.9733	0.4033	0.2283	0.9968	**0.0031**	0.3075
Anaerostipes	0.7437	0.9987	0.4703	0.7134	0.6364	0.8193
Akkermansia	0.2052	0.5247	0.0887	0.6036	**0.0017**	0.5867
*Otoolea*_134252	0.9572	0.8002	0.7011	0.1842	0.4148	0.0943
*Phascolarctobacterium*_A_40086	0.8572	0.8102	0.7211	0.1942	0.4348	0.0933
*Sarcina*_200052	0.9468	0.2371	0.7206	0.3183	0.1732	0.7263
*Clostridium*_R_135822	0.2194	0.7216	0.6277	0.6007	0.1497	**0.0085**
*Roseburia*_A_166204	0.1368	0.4603	0.7875	0.7833	**0.0015**	0.0974
*Parabacteroides*_B_862066	0.3618	0.1575	0.1647	0.0993	0.1558	0.7469
Anaerotruncus	0.7471	0.9275	0.4314	0.7518	0.5192	0.7351
*Odoribacter*_865974	0.9419	0.9903	0.4347	0.2260	0.2974	0.6835

*Mediterraneibacter*_A_155507	0.5546	0.6337	0.0928	0.2464	0.4459	0.6383
Cavicella	0.7892	0.6548	0.9884	0.6014	0.1301	**0.0036**
Allosphingosinicella	0.3736	0.3764	0.4308	0.8827	0.9406	0.1415
Granulicatella	0.3866	0.4979	0.0805	0.9208	0.4185	0.1925
*Blautia*_A_141780	0.2799	0.6180	0.6801	0.9547	0.4812	**0.0047**
Bilophila	0.5824	0.8100	0.7338	0.6389	0.5051	0.4654

Supplementary Table S4. Functional classification of bacterial genera included in the ecological analyses. Bacterial genera were assigned to functional categories based on manually curated evidence from published experimental studies and authoritative review articles. Because the ecological role of several genera is species- and context-dependent, multiple functional categories were assigned when supported by the literature. Representative references supporting each functional annotation are provided.

Genus	Functional classification	
Akkermansia	Mucin degrader / Commensal / anti-inflammatory/ SCFA indirect producers	DOI: 10.1099/ijs.0.02873-0; DOI: 10.1136/gutjnl-2021-326789
Agathobacter	SCFA producer (Butyrate) / anti-inflammatory	DOI: 10.1099/ijsem.0.000788
Alistipes	Multiple context-dependent functions (Commensal / Pathobiont) / SCFA producer	DOI: 10.1080/19490976.2019.1638722
Acinetobacter	Opportunistic / Pathobiont	DOI: 10.1128/CMR.00058-18
Actinomyces	Commensal (Mucosal resident)	DOI: 10.1128/JCM.01362-14
Anaerobutyricum	SCFA producer (Butyrate) / anti-inflammatory	DOI: 10.1016/j.syapm.2013.11.002
Anaerostipes	SCFA producer (Butyrate / Acetate) / anti-inflammatory	DOI: 10.1128/AEM.70.10.5810-5817.2004
Bacteroides	Multiple context-dependent functions (Commensal / Mucin degrader / Pathobiont) / SCFA producer	DOI: 10.1128/CMR.2.4.372; DOI: 10.1128/CMR.00008-07
Barnesiella	SCFA producer / anti-inflammatory / Commensal / Mucin degrader	DOI: 10.1128/IAI.00163-13
Bifidobacterium	Commensal / SCFA producer (Acetate / Lactate) / anti-inflammatory	DOI: 10.1038/gto.2011.19
Bilophila	Inflammation-associated taxon / Pathobiont	https://doi.org/10.1038/nature11225
Butyricicoccus	SCFA producer (Butyrate) / anti-inflammatory	DOI: 10.1111/jam.12297
Campylobacter	Opportunistic / Pathobiont	DOI: 10.1111/j.1574-695X.2011.00843.x
Citrobacter	Opportunistic / Pathobiont	DOI: 10.1128/IAI.76.10.4397-4405.2008
Clostridium	Multiple context-dependent functions (Commensal / Pathobiont / SCFA producer)	DOI: 10.5009/gnl.2013.7.5.485
Coprococcus	SCFA producer (Butyrate) / anti-inflammatory	DOI: 10.1099/ijs.0.02925-0
Dialister	Multiple context-dependent functions (Commensal / SCFA producer / anti-inflammation-associated)	DOI: 10.1111/prd.12023
Eggerthella	Multiple context-dependent functions (Commensal/ Xenobiotic metabolizer)	DOI: 10.1038/s41586-019-1333-1
Enterobacter	Opportunistic / Pathobiont	DOI: 10.3389/fmicb.2019.02468
Escherichia	Multiple context-dependent functions (Commensal / Pathobiont)	DOI: 10.1038/nrmicro2298
Faecalibacterium	SCFA producer (Butyrate) / Anti-inflammatory commensal	DOI: 10.1073/pnas.0800992105

Fusobacterium	Multiple context-dependent functions (Pathobiont / Inflammation-associated)	DOI: 10.1101/gr.133939.111
Gemella	Multiple context-dependent functions (Commensal / Pathobiont / Probiotic potential)	DOI: 10.1099/00207713-48-3-787
Hafnia	Multiple context-dependent functions (Pathobiont)	DOI: 10.3390/ijms21218252
Klebsiella	Opportunistic / Pathobiont	DOI: 10.1126/science.aal3919
Lactobacillus	Commensal / SCFA producer (Lactate) / Anti-inflammatory commensal	DOI: 10.1038/nrgastro.2014.157
Morganella	Opportunistic / Pathobiont	DOI: 10.1099/jmm.0.000305
Neisseria	Multiple context-dependent functions (Commensal / Pathobiont / Inflammation-associated)	DOI: 10.1099/mic.0.000030
Odoribacter	SCFA producer / Commensal	DOI: 10.1016/j.metab.2015.01.015
Parabacteroides	Anti-inflammatory commensal / SCFA producer / Mucin degrader	DOI: 10.3389/fmicb.2022.879555
Phascolarctobacterium	Anti-inflammatory commensal / SCFA producer (Propionate)	DOI: 10.3389/fmicb.2017.00673
Phocaeicola	Commensal / Mucin degrader / SCFA producer	DOI: 10.3389/fmicb.2019.02055
Prevotella	Multiple context-dependent functions (Mucin degrader / Commensal / Inflammation-associated)	DOI: 10.1038/nature10534; DOI: 10.7554/eLife.01202
Proteus	Opportunistic / Pathobiont	DOI: 10.1128/ecosalplus.ESP-0004-2017
Pseudomonas	Opportunistic / Pathobiont	DOI: 10.1093/femspd/fot052
Roseburia	Anti-inflammatory commensal / SCFA producer (Butyrate)	DOI: 10.1111/1574-6941.12384
Rothia	Commensal (Mucosal resident)	DOI: 10.1128/mSystems.00163-18
Ruminococcus	Multiple context-dependent functions (SCFA producer / Mucin degrader) / Pathobiont	DOI: 10.1074/jbc.M113.488639
Staphylococcus	Opportunistic / Pathobiont	DOI: 10.1056/NEJM199802123380707
Streptococcus	Multiple context-dependent functions (Commensal)	DOI: 10.1128/microbiolspec.GPP3-0007-2018
Sutterella	Inflammation-associated taxon	DOI: 10.1186/s13073-015-0199-7
Veillonella	SCFA producer / Cross-feeder	DOI: 10.1038/s41591-019-0488-4
